# New Insights into the Identity of the DFNA58 Gene

**DOI:** 10.3390/genes13122274

**Published:** 2022-12-02

**Authors:** Larissa Reis do Nascimento, Gleiciele Alice Vieira-Silva, João Paulo Fumio Whitaker Kitajima, Ana Carla Batissoco, Karina Lezirovitz

**Affiliations:** 1 Laboratório de Otorrinolaringologia/LIM 32, Hospital das Clínicas HCFMUSP, Faculdade de Medicina, Universidade de São Paulo, São Paulo 01246-903, Brazil; 2 Otorhinolaryngology Department, Faculdade de Medicina da Universidade de São Paulo, São Paulo 05508-060, Brazil; 3 Mendelics Genomic Analysis, São Paulo 02511-000, Brazil

**Keywords:** *CNRIP1a*, DFNA58, hereditary hearing loss, zebrafish, mice, expression gene analysis

## Abstract

Hearing loss is the most common sensory deficit, affecting 466 million people worldwide. The vast and diverse genes involved reflect the complexity of auditory physiology, which requires the use of animal models in order to gain a fuller understanding. Among the loci with a yet-to-be validated gene is the DFNA58, in which ~200 Kb genomic duplication, including three protein-coding genes (*PLEK*, *CNRIP1*, and *PPP3R1*′s exon1), was found to segregate with autosomal dominant hearing loss. Through whole genome sequencing, the duplication was found to be in tandem and inserted in an intergenic region, without the disruption of the topological domains. Reanalysis of transcriptomes data studies (zebrafish and mouse), and RT-qPCR analysis of adult zebrafish target organs, in order to access their orthologues expression, highlighted promising results with *Cnrip1a,* corroborated by zebrafish in situ hybridization and immunofluorescence. Mouse data also suggested *Cnrip1* as the best candidate for a relevant role in auditory physiology, and its importance in hearing seems to have remained conserved but the cell type exerting its function might have changed, from hair cells to spiral ganglion neurons.

## 1. Introduction

Hearing loss (HL) or deafness is one of the most common sensory defects in humans and usually interferes with social interaction, the development of language, interpersonal communication, speech, and learning, and might be associated with cognitive decline and depression [1,2]. According to the WHO (World Health Organization), about 466 million people worldwide (432 million adults and 34 million children) have HL and estimates predict a significant increase to more than 2.5 billion people affected by this disease by 2050 [3].

In developed countries, the frequency of early-onset (prelingual) HL with a genetic etiology is up to 60%. However, in developing countries such as Brazil, this frequency varies greatly among regions, due to the greater contribution of environmental factors in the less developed areas [4,5]. In 30% of genetic HL, it is syndromic, being accompanied by other clinical symptoms. HL is extremely heterogeneous, both in terms of clinical manifestation (age of onset, progression, audiological characteristics, and defect type) and etiology, with more than 120 genes and numerous environmental factors having been associated with it [6,7]. Regarding the age of onset, prelingual or postlingual HL refers to whether the HL manifested before or after language acquisition. Regarding the audiological characteristics, the severity (mild, moderate, severe, or profound), in addition to the affected sound frequencies, can vary greatly among genetic cases of HL. The defect might lie on the external and/or middle ear impairing sound conduction, named conductive, or in the inner ear or auditory nerve, named sensorineural, when sound transduction into electric impulses and its transmission to the central nervous system is impaired. Moreover, the underlying defect in genetic HL might also be mixed when both conductive and sensorineural components are present.

Genetic HL heterogeneity is also exemplified by all mendelian inheritance patterns it might exhibit, in addition to mitochondrial inheritance. Autosomal recessive is the most common pattern, present in about 80% of cases, and is usually associated with sensorineural prelingual [4]. Whereas autosomal dominant is the second most frequently observed in about 15–20% of the cases and is generally associated with postlingual progressive HL. Mitochondrial or X-linked inheritance each contribute to 1–2% [5,6].

The knowledge about the genes involved in nonsyndromic HL was mainly obtained through the genetic mapping of candidate loci by studying large families with many affected members that were clinically well characterized. To date, 73 loci associated with autosomal dominant nonsyndromic HL have already been mapped, but only 51 of them had their genes identified, illustrating the challenges of HL gene identification and functional validation. One of these genes that remains to be unraveled is the DFNA58, mapped in a Brazilian family [8]. Animal models have been crucial in the functional validation of HL candidate genes and in assisting the revealing of the auditory physiology complex gene network. The zebrafish has become an important nonmammalian model to uncover the challenges in genetic and molecular studies in HL imposed by the poor accessibility of the inner ear (IE). In this organism, the auditory system differs from that of mammals because it lacks the external/middle ears and the cochlea. However, its development and anatomy are similar to the IE of other vertebrates, and the sensory organs are composed of the same cell types present in the IE and in the vestibule of vertebrates: hair cells (HCs) that are innervated by neurons and supporting cells (SCs). These cell types are similar to their mammalian counterparts in both morphology and function and reside around the otoliths present in the IE and the neuromasts of the lateral line system [9].

The high homology with human genes involved in the signaling pathways for the formation and maintenance of the zebrafish auditory system allows investigation of the molecular and genetic mechanisms of differentiation, regeneration, death, and protection of HCs. Thus, various methodologies, ranging from cell transplants to genome edits, have been more easily performed on zebrafish embryos compared to murine models [10,11,12]. Finally, it merits highlighting the fact that, unlike mammals, fish can regenerate HCs throughout life as part of the physiological process of tissue growth and maintenance, due to the proliferation and transdifferentiation of SCs. Thus, the comparison of hearing-related genes in fish and mammals can lead to the discovery of key regeneration molecules and, with that, uncover new targets for a therapeutic intervention that aims to restore hearing [13,14].

Recently, the big data revolution, essential for open science, has increased the deposits in public repositories of gene expression datasets, which made it feasible to reanalyze the already validated initial results and their reuse for further analysis [15,16]. The integration and reanalysis of these diverse gene expression datasets have the potential to provide new insights into the biological mechanisms under study, encouraging reproducible science and bringing new perspectives, as it facilitates the elaboration and interpretation of hypotheses, pattern recognition, and data validation [17]. In addition, it constitutes an important tool to overcome financial and technical difficulties [14,18], where, for example, a gene expression dataset in which the initial study aimed to identify general patterns, with a more specific look it can bring new knowledge about the tissue or disease under study [19]. Furthermore, it promotes savings in money, time, animals’ lives, and work. To sum up, hypothesis-free data mining can provide the motivation and foundations for further experimental research [18]. Ultimately, and in many cases, the experimental data needed to gain new scientific knowledge may already be available and are just “waiting” to be investigated to answer open scientific questions. Thus, the search for differentially expressed genes (DEGs) in a given disease, or when comparing cell types from the same organ or tissue, has received increasing importance, since they can reveal new candidate genes, as well as the pathways of which they compose, which may be related to the pathophysiology of the disease, with the potential to reveal biomarkers and therapeutic targets [14]. In addition to the most known and used databases, such as NCBI, Gene Expression Omnibus (GEO) [20], Sequence Read Archive (SRA) [21], and ArrayExpress [22], there are already available in the literature several studies of transcriptomes of zebrafish larvae, which identified some specific genes of HCs (e.g., [23]), as well as transcriptomes of HCs and nonsensory surrounding cells of adult zebrafish [24,25]. Studies of mouse cochlear transcriptomes have also been published [26,27,28,29], as well as the cochlear sensory epithelium [30], other cochlear cell types [31] or comparing the different types of spiral ganglion neurons [32].

The DFNA58 locus, mapped by LEZIROVITZ et al. [8] to 2p12-21 in a large Brazilian pedigree with autosomal dominant postlingual progressive nonsyndromic HL, is one of those loci whose gene remains to be characterized. Exome NGS and Sanger sequencing unraveled a 200 Kb duplication, within the candidate chromosomal region (2p14), in affected individuals of this family, which includes two entire protein-coding genes (*PLEK* and *CNRIP1*), part of the *PPP3R1* gene, and about four long non-coding RNA (lncRNA) genes whose characterization also needs validation, *AC017083.1*, *LOC107985892*, *LOC102724389*, and *LOC101927723/AC015969.1-201* [33]. mRNA analyses of the duplication carriers’ blood showed the overexpression of the *CNRIP1* gene and two of the lncRNAs, as well as low abundant aberrant fusion transcripts. In situ hybridization and immunofluorescence studies performed in murine cochlea showed that the genes (*Cnrip1*, *Ppp3r1*, and *Plek*) and/or their proteins are expressed in spiral ganglion neurons, although *Cnrip1* has shown a higher labeling intensity, both in neonate and adult mice, particularly on type II neurons. Less remarkable expression was observed for the three genes in the organ of Corti and other cell groups of adult cochlea mice.

The observed overexpression of the *CNRIP1* gene and two of the lncRNAs, and possibly the fusion aberrant transcripts, may underlie the postlingual HL of the DFNA58 family [33]. However, as the biological role of these genes in hearing is still poorly understood, their expression profile characterization might pave the way for the revealing of the underlying pathophysiological mechanism.

To determine the expression profile of the *CNRIP1*, *PLEK*, and *PPP3R1* genes in the IE and, thus, contribute to the elucidation of the cause of HL in the DFNA58 family, a reanalysis of raw data from zebrafish and mouse transcriptomes, deposited at NCBI, GEO, and/or available in the Supplementary Materials of scientific papers, was performed to investigate the expression of their orthologues in these species. Furthermore, the levels of transcripts of orthologous candidate genes (*Cnrip1a*, *Cnrip1b*, *Ppp3r1a*, *Ppp3r1b*, and *Plek*) were evaluated using RT-qPCR in the auditory structures of adult zebrafish. Finally, given the promising results that highlight *Cnrip1a* as the best candidate to explain deafness in this family, its location in the sensory neuroepithelium in the zebrafish model of different ages was studied through in situ hybridization and immunofluorescence assays.

## 2. Materials and Methods

### 2.1. Whole Genome Sequencing

This study was approved by the Ethics Committee for the Analysis of Research Projects from the Institute of Biosciences and School of Medicine (both of the University of Sao Paulo). Written informed consent was obtained from all hearing-impaired individuals or their legal guardians, their relatives, and control individuals. To determine the exact insertion and extension of the DFNA58 duplication, whole genome sequencing was performed in a sample of an affected member of the family. The capture of the target regions of the human genome was done using custom genome probes, and next-generation sequencing on the Illumina platform. Alignment to the reference genome (GRCh38) was performed using the BWA (Burrows-Wheeler Aligner, https://bio-bwa.sourceforge.net/, accessed on 7 July 2021). Mitochondrial DNA sequencing was also analyzed. CNV identification was performed using in-house validated bioinformatics script.

### 2.2. Reuse of Zebrafish and Mouse Transcriptome Data

PubMed as well as Google searches were carried out using the keywords “transcriptome” and “inner ear” to find scientific studies that had conducted this type of investigation. The transcriptome data referring to RPKM (Reads Per Kilobase Million) or FPKM (Fragments Per Kilobase Million) or Mean Normalized Counts values of RNAseq were collected from the tables of Supplementary Materials and/or from tables available at NCBI and/or GEO obtained from studies already published. These papers did not analyze the candidate genes in the present study because it was not their focus. The RNA expression analysis data were taken from the following scientific papers: for *Danio rerio* [25,34,35] and for *Mus musculus* [30,31,36]. Aiming at characterizing the expression of the DFNA58 candidate genes in the IE and/or in populations of cells enriched with a specific cell type, such as HCs or their nonsensory surrounding cells, data from the genes *Cnrip1a*, *Cnrip1b*, *Ppp3r1a*, *Ppp3r1b*, and *Plek* were reused for zebrafish, while for *Mus musculus* the orthologous genes *Cnrip1*, *Ppp3r1*, and *Plek* were reanalyzed. In both studies, we also used data on the expression of genes already related to HL and/or expressed in the IE (*Slc1a3a*, *Eya4*, *Elavl4*, *Gsdmea*, *Gsdmeb*, *Pou4f1*, ^Gab1^, and *Mettl13*). As each study had its own parameters for the analysis of the already processed (nonraw) data, it was not possible to make comparisons between the numbers of each one of them, only to trace trends observed in more than one study, likewise the sites SHIELD and gEAR show data from different studies.

### 2.3. Animals

#### 2.3.1. Zebrafish 

Zebrafish specimens of the AB strain were used. The relationship between the ages of the animals and the tissues used for the RT-qPCR, in situ hybridization, and immunofluorescence analysis is described in Supplementary Table S1. All procedures were performed following the ethical and practical principles of using experimental animals, with the approval of the Ethics Committee on the Use of Animals of the Faculty of Medicine of the University of São Paulo (Research Protocol 089/17). For euthanasia, specimens were anesthetized with Tricaine (Sigma-Aldrich, St. Louis, MO, USA) at 3 mg/mL diluted in water (skin absorption) for 5–10 min. Then, they were placed in breeding tanks with water and immersed in Styrofoam-containing ice. After 15 min and with the confirmation of the animals’ deaths due to the reduction of body temperature, they were processed accordingly for RT-qPCR, in situ hybridization, and immunofluorescence analyses. For the RT-qPCR analyses, after euthanasia, dissections of the sensory organs utriculus + lagena and also of the brain were performed, following the protocol described by Einarsson et al. [37]. To be used as control reactions, the heart and swim bladder of the animals were also dissected. An incision was made in the animals’ abdomen and the heart and swim bladder were removed based on the morphology and anatomical position of these structures. Larval (5 dpf) and juvenile (30 dpf) specimens were also used to construct sense and antisense RNA probes for in situ hybridization analyses. For in situ hybridization and immunofluorescence, after euthanasia, the animals were immediately preserved in 4% PFA (Electron Microscopy Sciences, Hatfield, PA, USA) and stored until use at −20 °C.

#### 2.3.2. Mouse 

Whole cochleae harvested from male Balb/c mice (*Mus musculus*) were used, from neonates (up to three days of life) and mice about four weeks old. Animal work conformed to the Ethics Committee on the Use of Animals from the Hospital das Clínicas HCFMUSP, Faculdade de Medicina, Universidade de São Paulo-SP (0099/14, 100/14 e 101/14). Specific pathogen-free animals, without signs of external and/or middle ear infection or with ear malformations, visualized under the stereomicroscope, were included in the study. The whole cochlea was used for RNA extraction and obtaining protein lysates.

### 2.4. RNA Extraction and cDNA Synthesis

Whole animal and/or dissected tissues were transferred to a 2 mL microtube containing 800 µL of QIAzol (Qiagen, Hilden, Germany) and the tissue was homogenized with a rotator-stator (T10 Basic Ultra Turrax, Ika, Staufen, Germany). RNA extraction was performed with a miRNAsNasy Serum/Plasma Kit (50) (Qiagen, Hilden, Germany) according to the manufacturer’s instructions. To verify the concentration and purity of the extracted RNA, quantification was performed using the NanoDrop Technologies ND-2000c (Thermo Fisher Scientific, Inc, Waltham, MA, USA). cDNA was synthesized from 1µg of RNA with a SuperScript™ III First-Strand Synthesis System (Invitrogen, ThermoFisher Scientific, Waltham, MA, USA, EUA).

### 2.5. Quantitative Real-Time PCR (qPCR)

For the analysis of the transcript levels, we used, as a control, genes that are known to be related to hearing or HL and whose expression pattern in the mouse IE was similar to that observed for the genes *Cnrip1*, *Ppp3r1*, and *Plek* [33]. Thus, eight genes were selected: *Eya4* (DFNA10) [38] and *Dfna5* (*Gsdmea* and *Gsdmeb*) [39] due to high evidence of association with HL (https://panelapp.genomicsengland.co.uk/; accessed on 1 July 2021); *Pou4f1* and *Elavl4* due to high expression in sensory neurons on the statoacoustic ganglion of zebrafish, equivalent to the spiral ganglion in mice [31,40]; *Scl1a3a* because its expression is mostly in the IE of zebrafish and with a possible role in hearing in mice [40,41,42,43]; and *Gab1*, known to be an essential structure in the tyrosine kinase/HGF receptor pathway of the MET/MET/HGF proto-oncogene and responsible for the HL associated with DFNB26 and its modifier *Mettl1* [44]. The choice of these two genes had, as a parameter, the fact that the *CNRIP1* and *PPP3R1* genes are related to cancer in humans.

To quantitatively investigate the expression of the candidate genes of the DFNA58 locus by analyzing their mRNA (*Cnrip1a*-NM_001003607.2, *Cnrip1b*-NM_001327770.1, *Ppp3r1a*-NM_001327772.1, *Ppp3r1b*-NM_001004553.1, and *Plek*-NM_2. 1), as well as genes already related to HL and/or expressed in the IE (*Slc1a3a*-NM_212640.1, *Eya4*-NM_001282173.1, *Elavl4*-NM_001244600.1, *Gsdmea*-XM_005170077.3, *Gsdmeb*-NM_001001947.1, *Pou4f1*-NM_001312866.2, *Gab1*-NM_001326713.1, *Mettl13*-NM_001044769.4), RT-qPCR was performed using the dissected tissues of the IE that contained the neurosensory epithelium responsible for mechanotransduction (lagena + utricle). To compare the expression levels of these genes, other zebrafish tissues, such as the IE, heart, swim bladder, and brain, were used. Primers were designed with Primer-Blast (https://www.ncbi.nlm.nih.gov/tools/primer-blast/, accessed on 11 November 2020) (Supplementary Table S2).

PowerUP SYBR Green Master Mix 1X (Applied Biosystems, Thermo Fisher Scientific, Waltham, MA, USA) and 0.2 µM to 0.35 µM of each primer were used in each RT-qPCR reaction (Supplementary Table S2). Into the 48-well MicroAmp^®^ Fast Optical 48-Well Reaction Plate or MicroAmp^TM^ Optical 8-Tube Strip (both, Applied Biosystems, Thermo Fisher Scientific Waltham, MA, USA), 8 µL of the mix (SYBR master mix + primers) was added and 2 µL per well of cDNA at ~20 ng/µL. The qPCR runs were performed in the Step One equipment (Applied Biosystems, Thermo Fisher Scientific, Waltham, MA, USA) following the cycling conditions: activation at 50 °C for 2 min (1 cycle); activation of DNA polymerase action at 95 °C for 2 min (1 cycle); denaturation at 95 °C for 15 sec (50 cycles); and annealing and extension at 60 °C for 1 min (50 cycles). All RT-qPCR experiments were performed in duplicates, and in all of them the melting curve was performed at 98 °C after the end of the amplification to verify a single peak, demonstrating that the PCR is specific, and that it was very similar between different samples amplified with the same pair of primers. The specificity of each pair of primers was confirmed by sequencing the PCR products generated by the RT-qPCR of the zebrafish inner ear samples.

The amplification efficiency of each pair of primers was calculated using a standard curve of serial dilutions. The mathematical model proposed by PFAFFL [45], that takes into account the efficiency of the primers, was used in the calculation of the relative quantification. The following reference genes were tested: *Actb2*, *Ef1*, *Rps18*, *Sep15*, *Metap1*, *Rpl13*, and *Gapdh* [46,47]. Due to the reproducibility of efficiency and relative expression stability among different tissues/organs, the reference genes *Actb2* and *Rpl13* were selected, and their geometric means or medians were used.

Based on the Cts values (the higher the expression, the smaller the Ct), and taking into account the very high expression levels of some of the reference genes (Cts below 20), high expression (Cts between 20 and 27), median expression (Cts between 28 and 32), and low expression (Cts above 33), the other genes were analysed (Supplementary Table S3). The DeltaCt values were also obtained, which is the difference between the Ct value of the target gene and the median of two reference genes (Supplementary Table S3). As the Ct values of the reference genes are smaller because they are more expressed, negative values are obtained, and the closer to zero the greater the expression (the smaller the difference between the target and reference). Therefore, to facilitate the construction and visualization of the plots, the deltaCt was inverted to 1/deltaCt [25,48].

### 2.6. Statisical Anaylsis

To perform the statistical tests, the unpaired two-tailed Student’s t-test with Welch’s correction was used with the aid of the Graphpad Prism 9.02 software (2021) to determine whether the difference observed between the means of the various tissues was statistically significant and set as significantly different when *p* < 0.05. Normality was checked by the Shapiro–Wilk test.

### 2.7. In Situ Hybridization Analyses

A minimum number of 15 larvae (5 dpf) and one juvenile specimen (30 dpf) was established to be used in each experiment performed with *Cnrip1a*. The choice of these ages is because, at 5 dpf, the otic vesicle (OV) is fully developed and, therefore, easier to be visualized, and, at 30 dpf, the IE is completely formed. For each age, gene, and target probe, experiments were performed in duplicates to validate the expression pattern observed for each probe, both sense and antisense, and for each gene evaluated.

For the construction of the mRNA probes, primers were designed using the Primer3 computer package (https://primer3.ut.ee, accessed on 6 June 2019) containing the T7 promoter sequence upstream to its sequences (Supplementary Table S4). For the antisense probes, for each gene, the T7 promoter sequence was added at the beginning of the reverse primer, while for the sense probes, the sequence was added to the beginning of the forward primer. The designed sequences were submitted to the BLAST alignment tool (Basic Local Alignment Search Tool, https://blast.ncbi.nlm.nih.gov/Blast.cgi, accessed on 6 June 2019) to ensure the specificity of these segments to the target gene. While the antisense probes are intended for the analysis of gene expression, the sense is used as a negative control because it will not be complementary to the mRNA, since, in the cell, the mRNA is sense and, therefore, only the antisense probes will detect it.

To obtain the RNA probes (sense and antisense), the amplification reaction of the cDNA segments was performed in a final volume of 25 μL, containing 1X Platinum Buffer, 1.5 mM MgCl2, 0.24 mM dNTPs, 0.4 µM each of the forward and reverse primers, 1.25 U of Taq DNA polymerase Platinum (cat. 10966030, Invitrogen, Thermo Fischer Scientific), and 1 μg cDNA. The following cycle sequences were used on the Veriti 96 Well Thermal Cycler (Applied Biosystems, Thermo Fisher Scientific, Waltham, MA, USA): initial denaturation at 95 °C for 5 min, 10 denaturation cycles for 45 sec at 95 °C, annealing for 45 sec at 67 °C (decreasing 1 °C/cycle), extension for 1 min at 72 °C, followed by 24 cycles of 95 °C for 45 sec, annealing for 45 sec at 57 °C, extension at 72 °C for 1 min, and, final extension at 72 °C for 5 min. Finally, the specificity of the amplified fragments was verified by electrophoresis in a 2% agarose gel. Then, the amplified fragments were purified with the DNA Clean & Concentrator^TM^—5 kits (Zymo Research, Irvine, CA, USA) according to the manufacturer’s instructions. Purified PCR products were eluted with 11 μL of DNA Elution buffer followed by quantification in NanoDrop Technologies ND-2000c (Thermo Fisher Scientific, Inc, Waltham, MA, USA) to verify the efficiency of the purifications.

Transcription reactions of the mRNA probes were performed with a total volume of 20 μL containing 4 μL 1X T7 Buffer (Promega Corporation, Madison, WI, USA), 2 μL DTT (Promega Corporation, Madison, WI, USA), 2 μL DIG RNA Labeling Mix 10X (Roche, Basel, Switzerland), 40 U RNAse OUT Inhibitor (Thermo Fisher Scientific, Inc, Waltham, MA, USA), 40 UT7 RNA polymerase (Promega Corporation, Madison, WI, USA), and 1 μg of the purified cDNA, to synthesize 1 μg of each probe. The reactions were incubated at 37 °C for 4 h in the Veriti 96 Well Thermal Cycler and, in the end, the transcription efficiency of the mRNA probes was verified by electrophoresis in a 2% agarose gel. Then, purifications of the mRNA probes were performed using SigmaSpin™ Post-Reaction Clean-Up Columns (Sigma-Aldrich, St. Louis, MO, USA) according to the manufacturer’s instructions. Purified probe products were eluted with 20 µL of water. An amount of 1 μL of 5 mM EDTA pH 8.0 (C10H16N2O8) and 9 μL of RNAlater (Qiagen, Hilden, Germany) were added to each sample to prevent its degradation. After purification, samples were quantified on the NanoDrop Technologies ND-2000, and an aliquot of each sample was subjected to 2% agarose gel electrophoresis to verify the quality of purification.

For the preparation of the larvae and juvenile fish, they were fixed with 4% paraformaldehyde (PFA) (Electron Microscopy Sciences, Hatfield, PA, USA), incubated overnight at 4 °C, washed with PBST (PBS 1 X [H2O; Na2HPO4 10 mM; 1.7 mM KH2PO4; 136 mM NaCl; 2.7 mM KCL] + 0.1 mM Tween) and fixed in 100 Methanol (Merck, Darmstadt, GER). Then, for body depigmentation, they were incubated for 30–40 min in a solution containing 3% H2O2 and 0.5% KOH (both from Sigma-Aldrich, St. Louis, MO, USA) and washed with PBST. Permeabilization was performed by incubation with 10 mg/mL Proteinase K (Roche, Basel, Switzerland) at RT for 30 min. For the whole juvenile zebrafish, the concentration was 50 mg/mL and the incubation was 1 h. Then, to inhibit the action of proteinase K, the specimens were incubated with 4% PFA for 20 min at RT and washed with PBST. Incubations were carried out for 2–5 h in a dry bath at 70 °C with the hybridization solution (1M Citric Acid pH 6, 50% Formamide, 5 μg/mL Heparin, 5X SSC (Sodium Citrate Buffer), 500 μg/mL tRNA (Sigma-Aldrich, St. Louis, MO, USA), and 0.1% Tween 20, diluted in DEPEC Water or Milli-Q).

For the in situ hybridization reaction, the samples were incubated at 70 °C overnight in the hybridization solution containing 1 μL of the mRNA probe of each gene (antisense and sense) labeled with DIG-Dioxigenin (Roche, Basel, Switzerland). To avoid the unspecific labeling of probes that do not hybridize, the samples were washed for 10 min, under agitation, and at 70 °C with the following solutions: 3 X with HM (hybridization mix: Citric Acid 1M pH 6, Formamide 50%, SSC 5 X and Tween 20 at 0.1%) in the following concentrations of HM: 75%, 50%, and 25%; 1 X with 2 X SSC; 2 X with 0.2 X SSC. Then, three washes were performed at RT with 0.2 X SSC diluted in PBST, for 10 min each. In blocking, the samples were incubated for 3–4 h at RT with 2% sheep serum and 2 mg/mL bovine serum (Bovine Serum Albumin) (both from Sigma-Aldrich, St. Louis, MO, USA), the buffer was discarded and the samples were incubated with anti-DIG antibody 1:5000 (Roche Diagnostics, Indianapolis, USA) overnight at 4 °C with shaking, followed by washes of 5 min at RT with TBS (MgCl2 50 mM; NaCl 100 mM; 100 mM Tris HCL; 0.1% Tween 20) and subsequent incubation at RT with blue tetrazolium nitro chloride (NBT) at 225 μg/mL and 5Bromoo-4-chloro-3-indolyl phosphate (BCIP) at 175 μg/mL (both reagents from Sigma-Aldrich, St. Louis, MO, USA). The incubation time was monitored until the desired color intensity was obtained and the reactions were stopped. For this, the samples were transferred to 1.5 mL microtubes and subjected to three washes with stop solution (PBS pH 5.5, 1 mM EDTA, 0.1% Tween 20) for 15 min each at RT and with gentle agitation. Finally, the samples were incubated for 10 min at RT in the following concentrations of glycerol (Sigma-Aldrich, St. Louis, MO, USA), 25%, 50%, and 75%, diluted in PBS. The specimens were placed on slides and viewed under an S8APO stereomicroscope (Leica Microsystems, Germany), and the images were captured with the DF C450C camera (Leica Microsystems, Germany) with the Las V4.1 software (Leica Microsystems, Germany). The in situ hybridization protocol described above was based on that described by THISSE; THISSE [49].

### 2.8. Indirect Immunofluorescence

After fixation in 4% PFA at 4 °C overnight, the specimens were depigmented as described in the in situ hybridization protocol. Then, they were incubated with 10% sucrose (Sigma-Aldrich, St. Louis, MO, USA) for 2 h, and then, with 20% sucrose overnight at 4 °C. Then, they were washed twice with PBS for 5 min and stored in PBS at 4 °C until use.

For immunofluorescence, the larvae or whole fish were permeabilized with acetone (Sigma-Aldrich, St. Louis, MO, USA), previously chilled, for 20 min at 20 °C. Then, they were incubated at 4 °C overnight with a blocking solution consisting of 2% goat serum (Abcam, Cambridge, UK) and 1% BSA, and, subsequently, incubated at 4 °C overnight with primary antibodies diluted in a PBS buffer containing 1% BSA, using the following concentrations: Cnrip1 Rabbit 1:50 (kindly provided by Dr. Ken Mackie) and Phalloidin 1:50 (Thermofisher Scientific, A22287, Waltham, MA, USA). Subsequently, they were incubated with Alexa Fluor 488 (1:400) or Alexa Fluor 546 (1:300) secondary antibodies (Molecular Probes Thermo Fisher Scientific, Waltham, MA, USA) diluted in a PBS buffer containing 1% BSA for 4 h at RT. With 30 min left until the end of the incubation, DAPI (4’,6-diamidino-2-phenylindole) (Molecular Probes Thermo Fisher Scientific, Waltham, MA, USA) was added at a concentration of 1:1500. The specimens were transferred to hollowed-out glass slides containing 1% low gelling agarose (Sigma-Aldrich, St. Louis, MO, USA) to fix larval positioning and the slides were sealed with a coverslip and stored at RT until the capture of the larvae. Images were obtained using a confocal fluorescence microscope LSM 780 (Carl Zeiss, Oberkochen, BW, Germany) or LSM880 (Carl Zeiss, Oberkochen, BW, Germany) and the images were captured with the Zen program (Carl Zeiss, Oberkochen, BW, Germany). As for the immunofluorescence assays, larval specimens with 4 dpf were used. The antibody was provided by researcher Dr. Ken Mackie of Indiana University. This antibody was produced from an all human CRIP1a protein that has about a 60% similarity to the proteins encoded by the zebrafish genes (*Cnrip1a*/*Cnrip1b*). Therefore, with this antibody, it is not possible to distinguish whether the expressed protein is encoded by the *Cnrip1a* or *Cnrip1b*. The antibody used had its specificity validated by immunofluorescence in a cell line with an overexpression of CRIP1a and by competition with peptides in mouse retinal sections [50,51]

### 2.9. Western Blotting Cnrip1-Inner Ear Mouse

Protein lysates were obtained using an RIPA buffer (50 mM Tris-HCL pH 8, 150 mM NaCl, 50 mM sodium fluoride, 5 mM sodium orthovanadate, 2 mM EGTA) and protease inhibitor cocktail (ThermoFisher Scientific, Waltham, MA, USA) with a rotator-stator for disruption and tissue homogenization of the mice’s whole cochlea and brain (P7 and P28). Protein quantification was performed using the Nanodrop spectrophotometer (ThermoFisher Scientific, Waltham, MA, USA). Forty micrograms of protein in a sample buffer (2% SDS, 100 mM dithiothreitol, 10% glycerol) were subjected to SDS-PAGE. Western blotting was performed by submitting the samples to electrophoresis (14% SDS-PAGE) and electro-transferring proteins to a 45 µm nitrocellulose filter (BioRad, Hercules, CA, USA) for 16 h at 25 V. Transfer efficiency was observed after 1.5% Ponceau S staining. Filters were incubated for 1 h with 1% casein (Novagen, Germany), followed by 10 min in 3% hydrogen peroxide. Blots were incubated with primary antibodies for 1 h each, at room temperature. Antibody dilutions were in 2% immunoglobulin-free bovine serum albumin (BSA, Jackson Immuno Research Laboratories, West Grove, PA, USA) in TBS-T (20 mM Tris pH 7.6, 135 mM NaCl, 0.05% Tween-20). Rabbit anti-β-actin (Abcam7, Abcam, Cambridge, UK) was used as a reference endogenous gene at a final concentration of 1:10.000, and the rabbit Anti-CRIP1a was from Mackie’s laboratory (dilution1:1000). The blots were then washed in TBS-T and incubated with an HRP-labeled goat anti-rabbit secondary antibody (1:500) at RT for 1 h. The protein of interest was visualized using an enhanced chemiluminescence system (ChemiDocMP, Biorad laboratories, Hercules, CA, USA). Densitometry analyses were performed using ImageJ 1.38 e software (http://rsb.info.nih.gov/ij/) and Image Studio Lite (https://www.licor.com/bio/products/) to measure the intensity of the bands.

## 3. Results

### 3.1. The Duplication Is in Tandem between the PLEK and FBXO48 Genes

**Figure 1:**
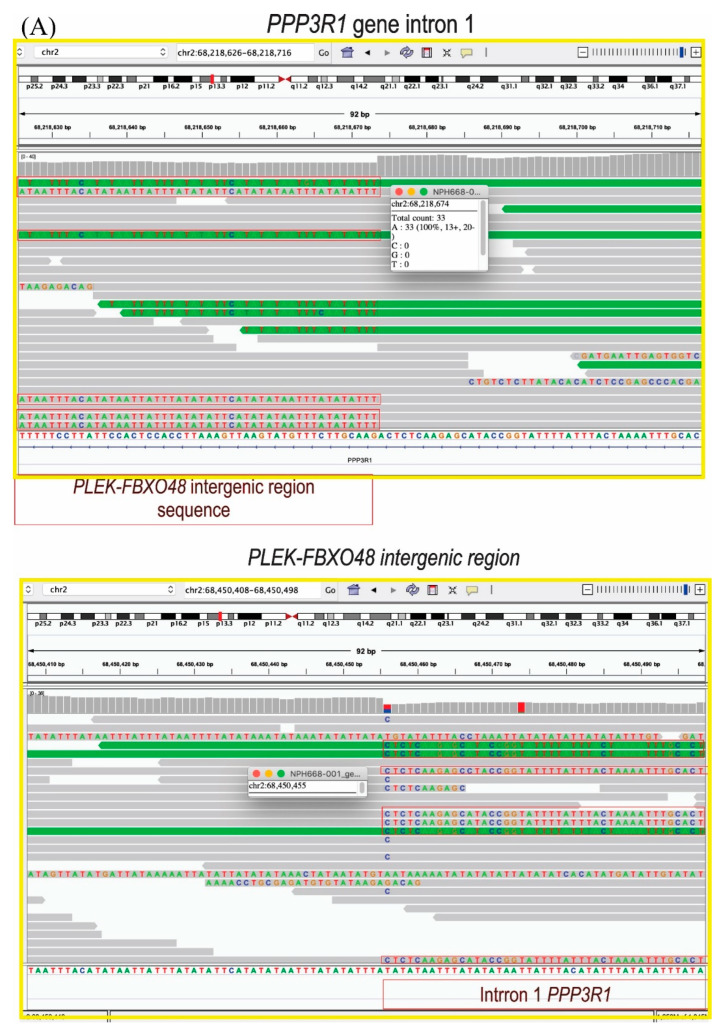

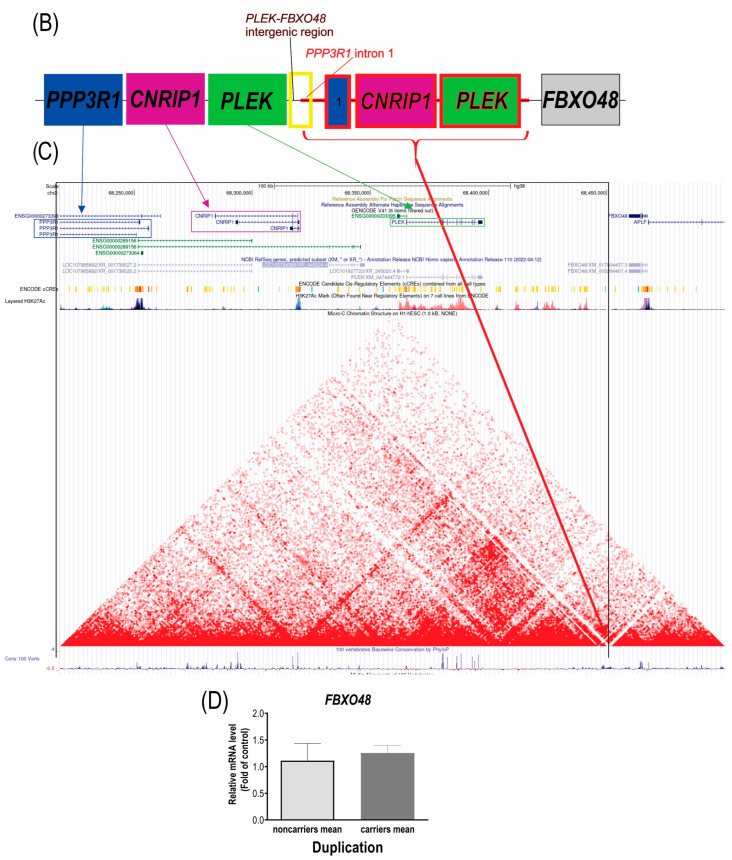
(**A**) Image of the WGS reads that showed the breakpoints 1 and 2 of the duplication and, contrary to the previous hypothesis, formulated based on the fusion transcripts, the duplication is in tandem, inserted in the breakpoint 2, in a repetitive region of (TA)n. (**B**) Scheme of the duplication (in red) showing its insertion site between the *PLEK* and *FBXO48* genes. (**C**) Analysis of insertion site and topological features. The black frame indicates the duplicated segment, the red arrow indicates the duplication insertion location on the border between the two domains; therefore, it does not interrupt either domain. (**D**) Gene expression analysis by RT-qPCR in the blood of the DFNA58 family members, comparing the means of normal-hearing noncarriers and hearing-impaired duplication carriers.

To verify the insertion position of the duplication and to refine its breakpoints, whole genome sequencing (WGS) of an affected duplication carrier was performed (Figure 1A). The results revealed that the duplication breakpoint 2 (BK2), in the 3’ intergenic region of *PLEK*, was at 68,450,455, within the range defined based on the previously used techniques, between the genomic positions 68,449,525 and 68,452,166. However, BK1 was more telomeric (68,218,674) than predicted by the other methods (range between 68,247,572 and 68,248,077, (Figure 1A–C)). In addition, the duplication was shown to be in tandem, inserted in BK2, immersed in a (TA) n-repetitive region, which is the intergenic region between the *PLEK* and *FBXO48* genes (Figure 1A–C). This kind of repetitive region generates instability and must have mediated this duplication. Therefore, the duplicate segment corresponds to the range chr2:68,218,674–68,450,455 with a total size of 231,782 bp and its insertion does not disrupt any gene.

The analysis of the insertion site and the topological domains showed that the duplication probably should only change the expression of the genes contained within it through gain of function by copy number, given that it did not interrupt any gene. Regarding the analysis of the topological domains, the duplication should not cause any rearrangement in the chromatin architecture, since the duplication contains the CTCF site responsible for the boundary of this domain (Figure 1C). The expression of the other gene that flanks the duplication insertion site, *FBXO48*, was also verified in the blood of the DFNA58 family members and we did not find any differences between the carriers and non-carriers of the duplication (Figure 1D).

### 3.2. Comparison by RT-qPCR between Reference/Control and Candidate/Target Genes within Each Zebrafish Tissue

**Figure 2:**
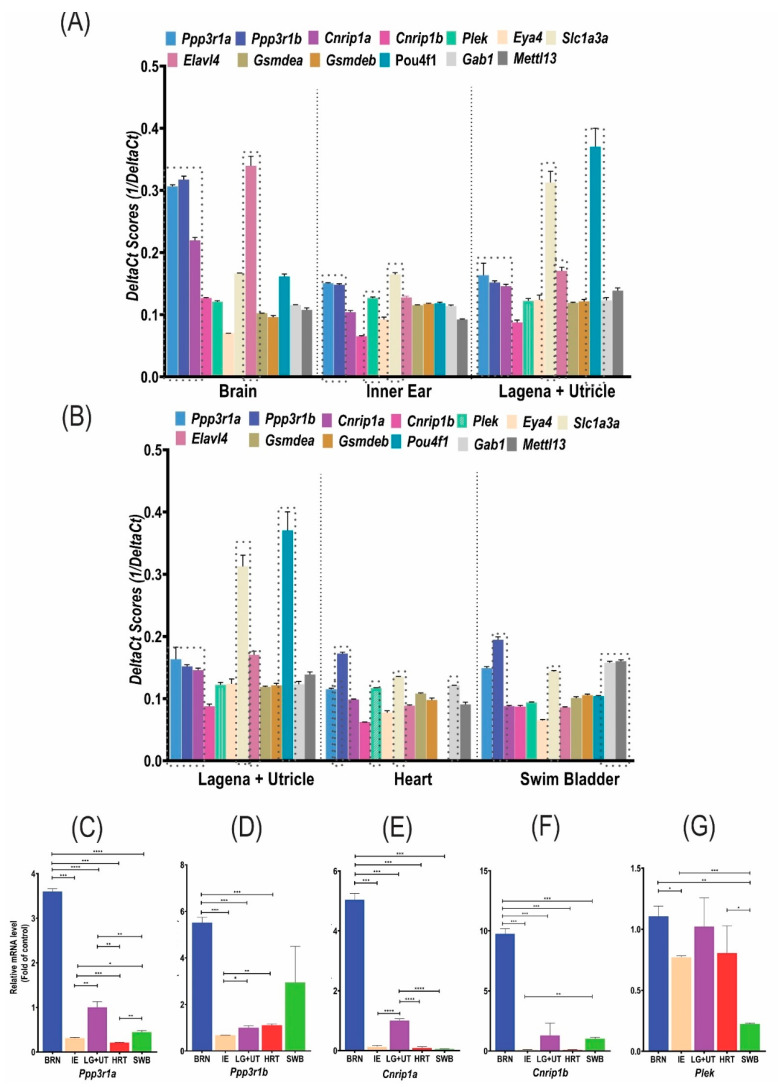

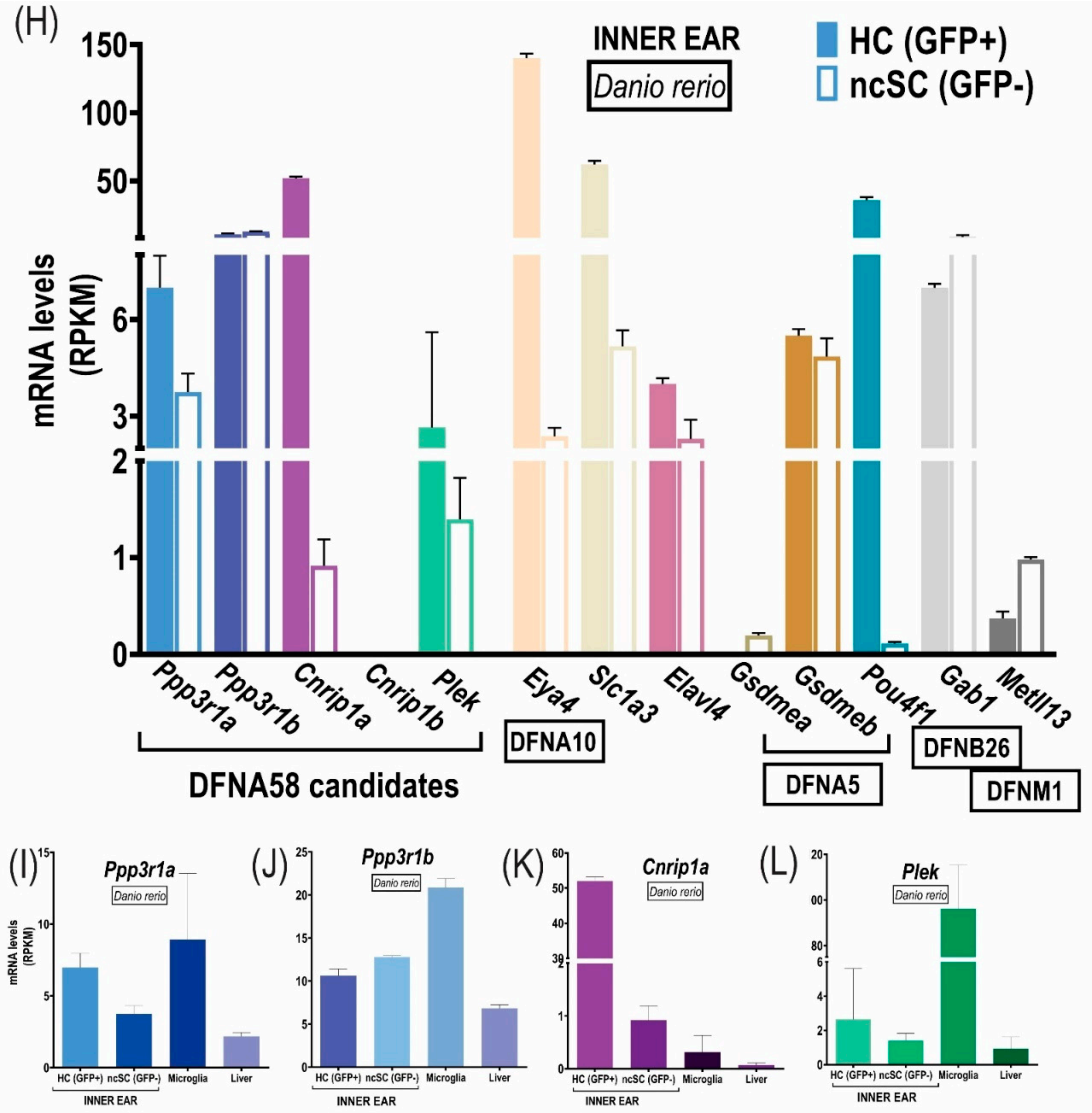
RT-qPCR and transcriptome data analysis of the DFNA58 genes and the reference/control genes. (**A**,**B**) Comparison of the level of expression of each gene in each tissue using 1/deltaCt values (RT-qPCR). (**C**–**G**) Relative quantification (delta-delta Ct of Pfafll [45]) regarding each DFNA58 gene (other genes in the Supplementary Figure S1) in different tissues of the adult zebrafish (lagena + utricle as the reference sample, the other tissues being fold of its expression). In (**H**–**L**), results of the reanalysis of data from transcriptomes, comparing the expression levels of genes from the DFNA58 locus and the reference genes regarding the zebrafish inner ear hair cells (HC) and their nonsensory surrounding cells (nsSC) in (**H**) and regarding the DFNA58 genes in the microglia [35], liver [34], and inner ear cells [25], as made available for public access by authors for reanalysis as part of open science. LG + UT: lagena + utricle; BRN: brain; HRT: heart; SWB: swim bladder; IE: inner ear. *,**,***,**** mean increasing levels of significance.

According to the RT-qPCR analyses, we observed that all protein-coding zebrafish orthologues (*Cnrip1a*, *Cnrip1b*, *Ppp3r1a*, *Ppp3r1b*, and *Plek*) to the DFNA58 duplication genes (*Cnrip1*, *Ppp3r1*, and *Plek*) are expressed in both the IE as a whole and the lagena + utricle neuroepithelium of adult zebrafish (2 years). In addition, their expression was observed in other zebrafish tissues such as the brain, heart, and swim bladder (Figure 2A,B, Supplementary Table S3). Genes already associated with hearing or deafness (mouse and/or zebrafish) were selected as controls/references for comparison and validation of the candidate genes’ results: *Eya4* (DFNA10), *Gsdmea* (DFNA5), *Gsdmeb* (DFNA5), *Pou4f1*, *Elavl4*, *Sclla3a*, *Gab1* (DFNB26), and *Mettl1* (DFNM). The Cts values were used and the levels of transcripts of each gene were quantified and compared to different tissues and each other (Figure 2A,B, Supplementary Table S3).

Based on the Cts values, it can be inferred that *Ppp3r1a*, *Ppp3r1b*, *Cnrip1a*, and *Plek* have a medium expression, while *Cnrip1b* has a low expression in these tissues (Supplementary Table S4). The DeltaCt values were used to compare the expression levels between the genes and tissues (Figure 2A,B, Supplementary Table S3).

#### 3.2.1. Inner Ear and Neuroepithelium (Lagena + Utricle) Expression

In the IE sample, as expected, the gene used as the control/reference, *Slc1a3a* (Figure 2A,B), showed the highest expression, followed by the candidate genes, *Ppp3r1a* and *Ppp3r1b*, which may indicate a possible role in hearing considering that the expression observed was higher compared to the known HL genes, *Eya4*, *Gsdmea*, *Gsdmeb*, *Gab1*, and *Mettl13*. The expression of the *Elavl4* and *Pou4f1* genes, known to be important in the neuronal process of hearing [31,43], were lower compared to the other genes. This could be due to an unexpressive portion of the neuronal tissue collected in our samples. *Plek* also showed expression in the IE, being slightly lower than that observed in *Elavl4*, while *Cnrip1a* showed a higher expression than *Eya4* and *Mettl13*, but lower than *Gsdmea*, *Gsdmeb*, and *Gab1*. Among all the DFNA58 genes studied in the zebrafish inner ear, *Cnrip1b* was the one with the lowest expression (Figure 2A,B).

Specifically analyzing the neurosensory epithelium (lagena + utricle), the expression of the *Pou4f1* gene, a neuronal marker with an important expression in the statoacoustic ganglion, stood out [31]. Then, in decreasing order of expression, there were *Slc1a3a* and *Elavl4*, followed by the candidate genes, *Ppp3r1a*, *Ppp3r1b*, and *Cnrip1a* (Figure 2A,B). It should be noted that, although the expression of these three candidate genes was lower than that observed in the IE, in the neuroepithelial structures, a probably higher expression was suggested by the results, compared to the other established HL genes, *Eya4*, *Gsdmea*, *Gsdmeb*, *Gab1*, and *Mettl13* (Figure 2A,B). *Cnrip1b* was also the one with the lowest expression, while *Plek* showed an expression level similar to well-established genes related to hearing. Furthermore, from the observation of the expression of these genes in the lagena + utricle of the adult zebrafish, these genes were more expressed in the sensory neuroepithelium compared to the whole ear, which may be suggestive of an expression exclusive to the sensory neuroepithelium.

#### 3.2.2. Other Tissues Expression

RT-qPCR analyses of the brain samples (Figure 2A,B) showed, as expected, a high expression of the *Elavl4* gene, followed by the candidate genes, *Ppp3r1a, Ppp3r1b*, and *Cnrp1a*, which may indicate an association of these three genes with neuronal cells, since the expression pattern observed was higher than for the *Slc1a3a* and *Pou4f1* genes, known for their expression in the IE and sensory neurons, respectively. *Cnrip1b* and *Plek* were the ones with the lowest expression, but superior to some of the genes associated with HL.

In the heart (Figure 2A,B), again, the *Ppp3r1b* gene showed the highest expression among the analyzed genes, followed by *Gab1*, *Slc1a3a*, *Plek*, and *Ppp3r1a*. The lowest expression gene was *Cnrip1b*. The *Pou4f1* gene transcript was not detected.

In addition, in the swim bladder (Figure 2A,B), the *Ppp3r1b* gene showed the highest expression, followed by the *Gab1*, *Mettl13*, *Ppp3r1a*, and *Slc1a3a* genes. The neuronal marker *Pou4f1* showed an expression similar to the HL genes (*Gsdmea* and *Gsdmeb*), while the candidate genes, *Plek*, *Cnrip1a*, and *Cnrip1b*, showed expression levels similar to *Elavl4* levels. In this organ, the lowest expression gene was *Eya4*.

### 3.3. Comparison by RT-qPCR of Each Candidate/Target Gene between the Neuroepithelium (Lagena + Utricle) and the Different Zebrafish Tissues

To quantify the levels of mRNA expression of each gene studied in the different tissues, our target tissue (lagena + utricle) was used as a normalizing reference sample through the deltaCT relative quantification method, proposed by Pfafll [45] (Figure 2C–G, Supplementary Figure S1, and Supplementary Table S5).

*Ppp3r1a* and *Ppp3r1b* (Figure 2C,D) exhibited a higher expression in the brain compared to all the other tissues analyzed, with an expression of 3.6X and 5.5X, respectively, higher than the lagena + utricle. A more significant differential expression in the lagena + utricle compared to the inner ear was observed for *Ppp3r1a* than for *Ppp3r1b* (0.31X and 0.67X, respectively). Whereas for *Ppp3r1a*, the lagena + utricle is second in terms of the highest expression; for *Ppp3r1b*, expression was more significant in the swim bladder and the heart.

*Cnrip1a* and *Cnrip1b* (Figure 2E,F) showed an even higher expression in the brain, compared to the lagena + utricle, of 5X, and 9.76X, respectively. The expression of both *Cnrip1a* and *Cnrip1b* in the lagena + utricle was also highlighted, because, although it was not as robust as that observed in the brain, it is considerably higher when compared to other tissues, especially in the IE (0.12X *Cnrip1a* and 0.07X *Cnrip1b*) (Figure 2E,F), suggesting the existence of a relevant gene function for these two specific tissues, the brain, and lagena + utricle.

*Plek* showed a similar expression in the lagena + utricle, brain, and heart, contrasting with the lower expression presented in the swim bladder and inner ear. However, there was a less expressive difference compared to the other candidate genes, indicating a more ubiquitous rather than differential expression between the investigated tissues (Figure 2G).

As for the other genes already consecrated as being important to hearing (Supplementary Figure S1 and Supplementary Table S5), *Slc1a3a* was, as expected, more expressed in the sensory neuroepithelium than in the other tissues, which includes both the IE (0.14X) and the brain (0.15X), a scenario similar to that observed for *Eya4* (brain 0.01X and inner ear 0.16X) and *Pou4f1* (brain 0.08X and inner ear 0.02 X). *Pou4f1* had such a high expression in the lagena + utricle that its expression in the heart was contemptible, an organ in which *Pou4f1* has been shown to have relevant expression during embryonic development [43]. Although there were no reports on its possible function in the IE of zebrafish, it is known that in mice it is associated with the development of spiral ganglion neurons [52]. Since the *Elavl4* gene is a marker of sensory neurons, it was not surprising that its expression in the brain was 7X higher than the lagena + utricle, but the higher expression in the inner ear (0.27X) compared to the heart (0.03X) and swim bladder (0.02X) was not expected. On the other hand, *Gab1* showed a higher expression in the lagena + utricle, but not significantly concerning the brain (0.87X) and the IE (0.78X). However, their expression in the swim bladder was almost 5X higher than in the lagena + utricle, similar to what was observed for its modifier gene *Mettl13* (3X), which might suggest that the pathway they share in hearing is also relevant for the swim bladder of the fish. Another interesting fact was that, in the heart, these expression levels were inverted; *Mettl13* had a low expression when compared to the lagena + utricle, as well as in the IE. Finally, the *Gsdmea* and *Gsdmeb* genes exhibited similar expression patterns between the lagena + utricle and the IE, while in the other tissues the expression of both was significantly lower.

### 3.4. Reuse of Transcriptome Data in Zebrafish

A reanalysis of zebrafish transcriptome data (RNA-seq) was performed regarding the DFNA58 genes’ expression and the same control/reference genes, obtained from three different studies (Supplementary Table S5, Figure 2H–L).

Barta et al. [25] used four adult fish (11–13 months), transgenic for the GFP gene under the *Pou4f3* promoter, to compare the expression profiles between 1000 GFP+ HCs with their stereocilia bundles and 1000 nonsensory GFP cells surrounding them (nsSC) from the lagena, saccule, and utricle (Figure 2H).

The microglial expression data came from Oosterhof et al. [35] that used 3-month-old neuro-NTR/mpeg1-GFP transgenic specimens (Figure 2I–L). As an expression control tissue, liver mRNA expression data from 3-year-old adult animals were used from Baumgart et al. [34] (Figure 2I–L).

Except for *Cnrip1b*, all the other candidate genes show relevant expression in the IE cells, whether HCs or nsSC (Figure 2H–L). In decreasing order of expression in the HCs, we have *Cnrip1a*, *Ppp3r1b*, *Ppp3r1a*, and *Plek* (Figure 2I–L). On the other hand, in the nsSC, in decreasing order of expression we have *Ppp3r1b*, *Ppp3r1a*, *Plek*, and *Cnrip1a* (Figure 2I–L). The most promising results appeared for *Cnrip1a* which was highly enriched in the HCs (RPKM 52X), differentially expressed, relative to the nsSC (RPKM 0.92X) similar to that observed for *Eya4*, *Pou4f1*, and *Slc1a3a* (Figure 2H). There were also differences in expression between the HCs compared to the surrounding cells, but not significant for *Ppp3r1a* (RPKM of 6.99 vs 3.72), *Ppp3r1b* (RPKM of 10.63 vs 12.80), and *Plek* (RPKM of 2. 65 vs 1.4) (Figure 2I–L).

Regarding the control genes, *Eya4*, *Slc1a3a*, and *Pou4f1* showed a high expression in the HCs, with *Pou4f1* being specific to this cell type. *Gsdmea* was not expressed in either cell type. The other genes did not show great differences in expression (Figure 2H). Comparing the expression of the DFNA58 genes observed in the IE, microglia, and liver, the robust expression of *Plek* and *Ppp3r1b* in the microglia compared to the total IE cells stood out (Figure 2I–L). DEGs between different cell groups of the same organ or tissue are more likely to play prominent roles. Using the table of these genes, created by Barta et al. [25], where a Fold difference (Log2) greater than 1.6 between the HC and nsSC indicates a differentially expressed gene, *Cnrip1a* and the reference genes, *Eya4*, *Slc1a3a*, *Pou4f1*, and *Mettl13*, were found to be differentially expressed (Supplementary Table S5).

Both the zebrafish transcriptome data and the RT-qPCR analysis highlighted the *Cnrip1a* gene in terms of its differential expression among the neuroepithelium cells, especially the HCs. *Ppp3r1a/b*, despite also being expressed in the sensory epithelia of the IE, had a more significant expression in the neuronal pathway accompanied by *Plek*.

### 3.5. In Situ Hybridization and Immunofluorescence of Zebrafish

**Figure 3:**
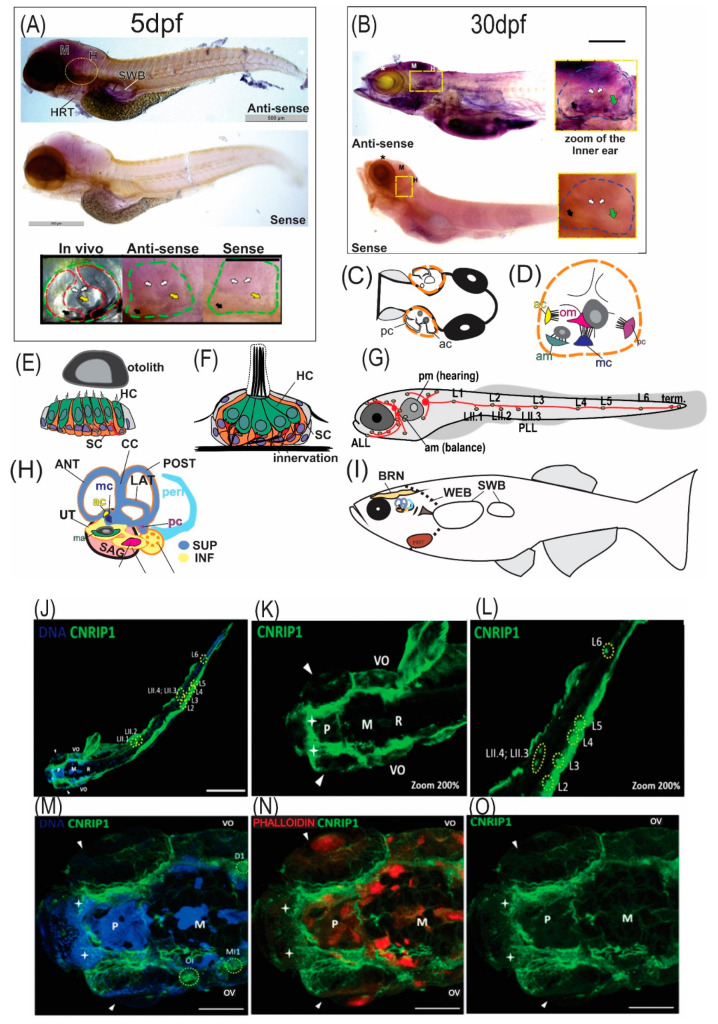
Expression and localization analysis of *Cnrip1* in the zebrafish. In (**A**,**B**) zebrafish in situ hybridization results in a lateral section of specimens with 5 dpf and 30 dpf, respectively. The telencephalon and forebrain (*) and midbrain (M) and hindbrain (H) brain regions, and heart (HRT) are indicated. The area delimited in yellow dotted in the larger figure and zoomed in on the smaller figure, refers to the otic vesicle (OV) in (**A**) and IE in (**B**). In both figures, at zoom (5X magnification) the OV dotted in green is shown; in red, the semicircular canals; yellow arrow: posterior saccular otolith; black arrow: utricular anterior otolith; white arrows: position of the semicircular canals. Scale bar: 500 μm (5 dpf) and 100 μm (30 dpf). (**C**–**I**) Schematic representation of the auditory sensory organ of zebrafish in larval (OV) and adult (IE) stage 5, as well as line lateral (LL) and its neuromasts. (**C**) Dorsal view of the head region with eyes in black. The OV and the anterior (CA) and posterior (PC) canals are represented by the black outlines; dotted orange line: the medial wall of developing otocyst; gray: otoliths. (**D**) Representation of the OV of a larva and the epithelial structures around which the semicircular canals are formed. (**E**) Cross section of IE neuroepithelium (anterior macula): HCs in green, SCs in orange, and the apical part of the epithelium is bathed by endolymph. (**F**) Longitudinal diagram of a neuromast showing the different cell types that compose it, quite similar to the IE. (**G**) Schematic representation of a larva with the location of the anterior (ALL) and posterior (PLL) LL; orange/green: arrangement of neuromasts; red: ganglia that receive the innervations from the neuromasts of each LL. (**H**) Representation of the IE of adult zebrafish. (**I**) Side view of an adult zebrafish. The schematic representations in the figures were designed/drawn taking as models/references the illustrations from [9,53,54,55,56]. (**J**–**L**) Immunofluorescence of a zebrafish specimen (4 dpf) with anti-cnrip1 labeled in green and cell nucleus in blue (DAPI). (**J**) Top/front view of the full extent of the specimen. Scale bar: 500 μm. (**K**) Zoom (200 x) of the head region. Highlighted are the brain regions M, P, and R, the OV, and the olfactory bulb (white star). (**L**) Zoom (200 x) of the final portion of the tail, highlighting LLP and its neuromasts (yellow circles). (**M**–**O**) Superior dorsal view of the head. In red, we have the phalloidin labeling showing the actin filaments. White arrow: eyes. Scale bar: 100 μm. Images were analyzed with a Zeiss LSM 780 confocal microscope and captured with ZEN software. VO: otic vesicle; LL: lateral line; BRN: brain; WEB: Weber’s ossicles; IE: inner ear; SWB: swim bladder; HRT: heart; MA: anterior macula; LAG: Lagena; UT: utricle; SAC: saccule; SAG: statoacoustic ganglion; mc: medial crest; Cp: mc posterior crest; pm: posterior macula; ANT: anterior semicircular canal; POST: posterior semicircular canal; CC: central semicircular canal; LAT: lateral semicircular canal; SUP: upper pars; INF: lower pars.

Given that both RT-qPCR and transcriptome analysis indicated the *Cnrip1a* gene as the best, as well as a promising candidate, a better location of its expression according to the IE structures was pursued. At 5 dpf, in situ hybridization labeling for *Cnrip1a* was observed in the brain regions (telencephalon, forebrain, midbrain, and hindbrain), liver, intestine, swim bladder, and pancreas. However, no labeling was detected at the otic vesicle for the *Cnrip1a* gene in this larval stage (Figure 3A). In the juvenile specimen of 30 dpf, in addition to the regions already identified in the larval stage, expression was also observed in the IE, from the superior region (semicircular canals) to the inferior region (saccule, utricle, and lagena) (Figure 3B). To facilitate the identification of the labeled structures, schematic representations of the IE and its developmental precursors, in addition to the lateral line, a sensory organ that contains the same cell types as the IE neuroepithelium, were shown in Figure 3C–I.

Regarding the immunofluorescence assays, due to the great similarity of the *Cnrip1a* and *Cnrip1b*-encoded proteins and the available antibodies, it was not possible to use a specific anti-Cnrip1a antibody. In Figure 3J–O, it is observed that the anti-Cnrip1a/b antibody labeled the neuronal network located throughout the body and head of the specimen. In addition to the expression in the peripheral sensory innervation, the proteins were also detected in the innervation of the otic vesicle, eyes, and the network of the neuromasts. Based on the identification of the neuromasts along the lateral line and head of the larval specimens, the schematic representation characterized by Haehnel-Taguchi et al. [57] as shown in Figure 3G, it was also possible to observe the expression of the proteins encoded by the *Cnrip1a*/*Cnrip1b* genes in the 4 dpf larvae in the head and lateral line neuromasts.

### 3.6. Expression Analyses in Mice

**Figure 4:**
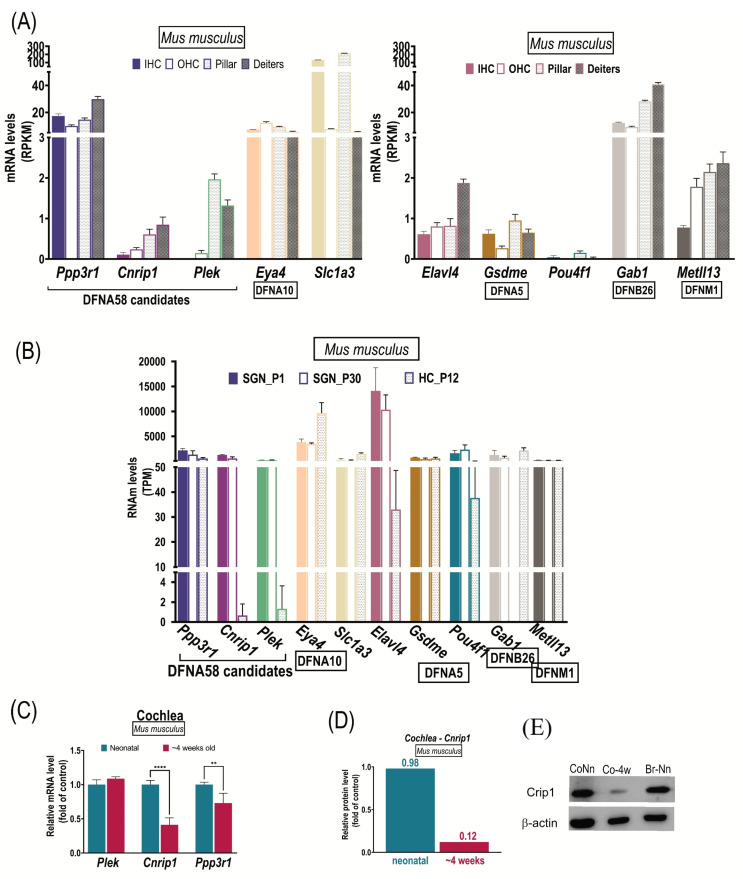
Reanalysis of transcriptome data from different studies. (**A**,**B**) as made available for public access by authors Li et al. [30], (**C**) Li et al. [31] for reanalysis as part of open science. (**D**) RT-qPCR of whole murine cochlea, (**E**) Western blotting of whole murine cochlea.

Among the mouse transcriptomes studies reanalyzed regarding our candidate genes, Hertzano et al. [36] characterized the gene expression profile of eight cochlear cell types, separated with flow cytometry, using an expression array containing 24,000 RefSeq genes annotated at the time and more than 7000 predicted genes. They obtained 3000 genes differentially expressed between the different cell types, among which only *Cnrip1* appeared (neither *Ppp3r1* nor *Plek*), as well as our following reference genes, *Slc1a3* and *Eya4*. Among the tissues analyzed, *Cnrip1 showed* a higher expression, in decreasing order: mesenchymal, neuronal, blood, and sensory. Whereas *Slc1a3* displayed a higher sensory than neuronal expression. The other studies, here reanalyzed, used RNA-seq analyses to compare the expression levels between the hair cells (inner hair cells vs. outer hair cells) and between the supporting cells (Deiters vs. Pillars) of adult mice [30] or between the spiral ganglion neurons of mice of different ages [31,32]. In Figure 4A–D, the RPKM/TPM plots displayed were constructed with the studies mentioned above, regarding the DFNA58 candidate genes (*Cnrip1*, *Ppp3r1*, *Plek*) and the control/reference genes (*Eya4*, *Gsdmea*, *Gsdmeb*, *Pou4f1*, *Elavl4*, *Sclla3a*, *Gab1*, and *Mettl13*).

The highest expression was detected for *Ppp3r1*, followed by *Plek* and *Cnrip1*. For *Cnrip1*, no great expression was observed in the inner (RPKM = 0.11) or outer (RPKM = 0.24) hair cells, being more enriched in the supporting cells Deiters (RPKM = 0.85) and Pillars (RPKM = 0.61). *Ppp3r1* showed the highest expression, but not significantly different among all cell types (IHC RPKM = 17.34, OHC RPKM = 9.95, pillar cells RPKM = 14.64, and Deiters cells RPKM = 29.84). *Plek*, in turn, had a higher expression in the supporting cells (pillars RPKM = 1.97, Deiters RPKM = 1.32) compared to the hair cells (IHC RPKM = 0.0, OHC RPKM = 0.15) (Figure 4A, Supplementary Table S6, data from Li et al. [30]). Although there were differences in the expression levels between the cell types studied in the cochlea (supporting cells and hair cells), none of the DFNA58 genes (*Cnrip1*, *Ppp3r1*, and *Plek*) showed a significant differential expression (Fold difference of 1.6X or more). The same pattern was observed for some of the studied reference genes, except for the *Slc1a3* gene, which was the only one that was revealed to be differentially expressed in the IHC compared to the OHC (2X Fold difference), as well as in the Pilar cells compared to the Deiters (Fold difference of 5.27X) (Figure 4B, Supplementary Table S6, data from Li et al. [30]).

As for the transcriptome data from Li et al. [31], in the SGN, the genes with the highest expression in descending order in P1 were: *Elval4*, *Eya4*, *Ppp3r1*, *Pou4f1*, *Cnrip1*, and *Gab1*; and in P30: *Elval4, Eya4, Pou4f1, Ppp3r1, Gab1, Cnrip1,* and *Gsmde/Dfna5*. *Plek* and the other deafness/hearing genes showed very low levels of expression at both ages (Figure 4B). *Cnrip1* maintained appreciable levels of expression at all ages in the SGN, being higher in P1 (TPM = 1279), both concerning the embryonic period (TPM = 518) and the period before the onset of hearing (P8/TPM = 893 and P14/TPM = 660), and the adult period (P30/TPM = 585), which is different from what was observed for the hair cells, with a low expression detected in P12. In the P8 glia (TPM = 322), it also showed appreciable expression, but lower than the SGN (TPM = 893) (Figure 4B). *Ppp3r1* was expressed both in the HC (P12/TPM = 565) and in the glia (P8/TPM = 999) and for different ages in the SGN (E15.5/TPM = 592, P1/TPM = 2191, P8/TPM = 2927, P14/TPM = 1187, and P30/TPM = 1339), although differentially higher in P1 (Figure 4B). *Plek* showed reduced expression in the glia (P8/TPM = 1.33) and lower in the HC at P12, and, despite having a higher expression in the SGN at different ages, it is lower when compared to *Cnrip1* and *Ppp3r1* (Figure 4B).

To sum up, the data of mouse inner ear transcriptomes also showed that the three DFNA58 candidate genes are expressed in spiral ganglion neurons, present but less significant in the sensory or supporting cells of the cochlea, and that the expression levels of *Cnrip1* and *Ppp3r1* are higher than that of *Plek* in these cells, differences that fall with age. Thus, the difference is significant for *Ppp3r1* concerning *Plek* in P1 (3.5X) and P30 (2.7X) and for *Cnrip1* concerning *Plek* in P1 (2.5X), an expression similar to that observed by Lezirovitz et al. [33] regarding in situ hybridization results.

To corroborate the data obtained with the reuse of the transcriptomes, RT-qPCR analyses were performed to quantify the transcripts of the *Cnrip1*, *Ppp3r1*, and *Plek* genes in the murine cochlea, neonatal period, and at about four weeks old. Among the three genes, a difference in the transcripts’ levels was observed, but, while *Plek* tended to display increased expression with age, *Ppp3r1*, and *Cnrip1* tended to decrease, and for these last two, this difference is statistically significant, more relevant for *Cnrip1* (Figure 4C). Accordingly, the protein levels of *Cnrip1* decreased with age (Figure 4D).

## 4. Discussion

Since the DFNA58 locus mapping [8] and the identification of its candidate genes, *CNRIP1*, *PPP3R1*, *PLEK*, and uncharacterized lncRNA genes [33], efforts have been focused on revealing their participation in autosomal dominant HL. In the previous paper, their expression in the patients’ blood and in the murine inner ear highlighted *CNRIP1* and two of the lncRNA genes as the best candidates, but the others were not ruled out [33]. Thus, a long way still remains to be able to conclude which one, or if more than one, is related to the pathophysiology of hearing loss in this family.

### 4.1. No Evidence of Additional Genes Affected by the Duplication

In the paper describing the DFNA58 duplication, the pieces of evidence from the fusion transcripts detected in the carriers’ blood suggested that its insertion position could be at 3’ downstream of the wild-type location of the *PPP3R1* gene. Here, we determined that its insertion position was in an intergenic region (between *PLEK* and *FBXO48*) and without the disruption of the chromatin topological domains. Thus, regarding the protein-coding genes, there was no evidence to support a direct effect on *PPP3R1* expression, given that neither a complete duplicated copy nor a disruption of its wild-type copy is present. These findings should refine the candidate protein-coding genes to *CNRIP1* and *PLEK*. However, given the existence of lncRNA in the duplication segment, without murine or zebrafish orthologues, *PPP3R1* remained a possible indirect candidate through its expression modulation by one of these lncRNA genes. The *Ppp3r1* gene has three different transcripts, each encoding proteins with different lengths (170 aa, 189 aa, and 160 aa), but also differing regarding their exon 1. Exon 1 from all three transcripts is involved in the duplication and contains the promoter. Exon 1 of the 170 aa transcript has only the initiation codon, exon 1 of the 160 aa has only UTR, but the exon 1 of the 189 aa has a few coding bases. In case these exons 1 were transcribed, nonsense-mediated decay should probably eliminate them as aberrant transcripts, avoiding a dominant-negative effect.

Thus, it was possible to conclude that it was not the insertion position of the duplication that allowed the PCR detection of the *PPP3R1*-*CNRIP1* fusion transcripts, as described in the previous paper by Lezirovitz et al. [33]. Another hypothesis might be that these aberrant transcripts were circular, as seen in several types of cancer, allowing their amplification using primers in opposite directions. The duplication first caused the juxtaposition of gene sequences that would otherwise be distant or in another order, and, secondly, the transcription of mature fusion mRNAs [58]. Guarnerio et al. [59] hypothesized that unrelated intronic sequences distant from the relocated genes would also have been juxtaposed. As a consequence, complementary repetitive sequences (e.g., Alu-sequences) could be placed close enough to favor new back-splicing events during the RNA maturation process, which would result in the generation of aberrant RNAs [60,61,62,63].

### 4.2. CNRIP1, PPP3R1, and PLEK Function Could Potentially Be Important for Hearing

Considering the plethora of functions already described concerning deafness genes and the functions of the three candidate genes, all three should be considered good candidates.

The Human *CNRIP1* gene encodes two protein isoforms, CRIP1a (164 aa) and CRIP1b (128 aa), which are identical from amino acids 1 to 110, differing from that position. CRIP1a is conserved in vertebrates [64], while CRIP1b is restricted to certain primates [65]. Both human isoforms are known for their interaction with the cannabinoid receptor 1 (CB1 or CBR1), which belongs to the family of G protein-coupled receptors, with the primary function of transducing extracellular stimuli into intracellular signals [66]. CRIP1a suppresses the tonic inhibition of voltage-gated calcium channels mediated by the CB1 receptor, whereas CRIP1b does not have this effect [67]. It should be noted that different studies have sought to identify other possible roles of these proteins in cell signaling and synaptic plasticity [66,68,69,70]. In zebrafish, a teleost fish, there are two orthologs to the human *CNRIP1* gene derived from a genomic duplication, *Cnrip1a* (chromosome 1, 1420 bp) and *Cnrip1b* (chromosome 13, 1015 bp). The two paralogous genes (*Cnrip1a* and *Cnrip1b*) show a 72% similarity between them in the coding region, resulting in a 93% similarity between the proteins, with differences mainly in the 28 amino acids of the N-terminal region. The proteins encoded by both orthologs are most similar to the human CRIP1a protein [64] [BLAST/NCBI]. Suppression of this gene expression has been reported in numerous types of cancer [71].

Calcineurin B (Cnb1), encoded by the *PPP3R1* gene, is a calcium-dependent serine/threonine phosphatase protein that is stimulated by calmodulin, which gives it sensitivity to calcium. It has already been observed in the cochlea, among other organs, that excess calcium causes cell death through a series of pathways that include calcineurin [72,73,74]. A study of survival-associated lung adenocarcinoma gene signatures found *PPP3R1* as one of 10 survival-associated genes, exhibiting an inverse association between mRNA levels and risk of death; that is, the higher the expression, the lower the risk of death. Furthermore, by RNA-Seq, a fusion between *PPP3R1* and *CNRIP1* genes was observed both in noninvolved tissue and in lung adenocarcinoma tissue [71]. While in humans, the *PPP3R1* gene has 3023 bp and its protein 170 aa, in zebrafish the homolog *Ppp3r1a*, located on chromosome 13, has 1203 bp and a protein of 175 aa (Ensembl and Blast/NCBI). *Ppp3r1b*, on the other hand, is located on chromosome 1, has 2795 bp, and its protein is 170 aa (ensembl.org). Both zebrafish proteins show about a 99% similarity with the human protein.

In humans, the *PLEK* gene has 32,281 bp that encodes pleckstrin, a 350 aa protein that constitutes the main substrate of protein kinase C in blood platelets in platelet activation [75,76]. In zebrafish, the *Plek* ortholog is located on chromosome 13, has 1902 bp, and has a protein with 352 aa (NCBI). Human pleckstrin shows about a 67% similarity to the zebrafish protein (https://blast.ncbi.nlm.nih.gov/Blast.cgi10, accessed on 6 June 2019). The role of pleckstrin is pointed out in several pathways of cellular interaction, among them the phosphorylation and heterologous desensitization of β-adrenergic receptors. It has already been observed that it can inhibit phosphoinositide hydrolysis induced by activating agonists of G protein-coupled receptors and growth factor receptors [77,78]. Furthermore, its overexpression in primary or transformed cells leads to an alteration in lamellipodia-like pseudopodia mediated by the organization of the actin cytoskeleton [79]. Lamelipodia resemble HCs stereocilia and their product interacts with radixin that has been associated with HL in humans and mice [79,80].

### 4.3. Expression Analyses in the Inner Ear

The genes associated with hearing loss in humans and mice show varied patterns of expression. There are those genes whose expression is practically exclusive to the inner ear. In other cases, they are ubiquitously expressed in many different tissues, although they may have some specific function in the cochlea; or there is a redundancy of function with other genes in other tissues, but not for the cochlea. Current studies on genetic hearing loss often report that these genes are highly expressed in the brain, as hair cells share similar characteristics with neurons, in addition to the presence of the neurons themselves that innervate the hair cells, whose cell bodies form the spiral ganglion [81]. In zebrafish, something similar occurs, where the sensory hair cells present in their auditory organs make up the neuroepithelium [10].

Generally, our expression data regarding the variety of tissues where these genes have been described are in agreement with the literature and databases (Supplementary Table S7). Differently expressed genes between different cell groups of the same organ or tissue are more likely to play prominent roles. The high expression identified in the brain of the *Cnrip1a/b* and *Ppp3r1a/b* genes supports a more relevant expression in neuron-rich tissues. The transcriptome reanalysis validates the RT-qPCR, in which the *Cnrip1a* gene, orthologous to *Cnrip1*, showed a significantly differential expression being enriched in the hair cells. Even though the *Ppp3r1a* gene also showed a higher expression in the hair cells, it was not as remarkable. Besides, unlike the *Cnrip1a* gene which was more expressed in the sensory epithelium of the lagena + utricle, *Ppp3r1a/b* were shown to be expressed in a wider variety of tissues, suggesting some role in hearing, although possibly similar to other tissues with an important neural component, but not related to cellular specializations typical of auditory physiology. Finally, the *Plek* gene that did not show significant expression levels in the individual and/or inner ear sensory epithelium did not suggest an important pattern in hearing.

In situ hybridization in zebrafish is a widely used method in the studies of candidate genes for hearing loss, because of the transparency of the specimens, as well as the easy visualization of the otic vesicle at stage 5 dpf and the complete development of the inner ear only at 30 dpf, which provide quick answers to questions related to whether or not the candidate gene is expressed in the target tissue. This application of in situ hybridization was very well exemplified in the study by [82] in which the candidate gene *Ncoa3* showed a large expression practically restricted to the sensory neuroepithelia. Our in situ hybridization analyses evidenced the diversity of organs in which *Cnrip1a* is expressed, in agreement with what was observed by Fin et al. [64]. They described the expression in the brain, heart, and swim bladder between 1 to 3 dpf. Our in situ hybridization and immunofluorescence data are also in agreement with those observed by Oltrabella et al. [83], who reported the conservation of *Cnrip1* gene expression in the neuronal network among different vertebrate species and also with what was described by Fin et al. [64], whose expression of *Cnrip1a* was evidenced in the regions of postmitotic neurons, covering the brain, neuromasts, and possible ganglia of the head region. The authors performed in situ hybridization assays from embryos with 10 hpf to 48 hpf. In this study, Fin et al. [64] also analyzed the labeling for *Cnrip1b* and observed that, between the developmental stage of 24–48 hpf, there was a divergence in the expression of *Cnrip1b* compared to that observed for *Cnrip1a*. Therefore, the authors concluded that both genes have a differentiation in terms of their expressions, suggesting that they could have different functions.

Through the reuse of transcriptome data from mice, we observed that *Cnrip1* and *Plek* in the hair cells presented a differential expression in comparison to their neighbors, being higher in the latter. The *Cnrip1* gene already stood out in terms of expression in the spiral ganglion neurons, where it was found to be differentially expressed between the two types of neurons (higher in type II), corroborating all the findings published in Lezirovitz et al. [33]. The differential expression between type I and type II neurons, being more abundant in type II neurons, as verified in Lezirovitz et al. [33], was also identified for the reference genes, *Gsmde*/*Dfna5* (0.267294349119519) and *Pou4f1* (0.00242505110388543) [32]. Interestingly, all three expressed at higher levels in type II neurons than in type I, which corresponds to 5% and 95% of SGN neurons, respectively. Furthermore, *Cnrip1* also appeared as one of the differentially expressed genes among the different cell types of the inner ear identified by HERTZANO et al. [36] in Cluster 6. This cluster is composed of genes that show a high expression in the sensory epithelium and a high relevance in the gene ontology of the extracellular matrix. Of the hearing loss genes that fell into the same cluster as *Cnrip1*, we have the *Coch* that encodes cochlin, a component of the extracellular matrix that is highly abundant in the cochlea and vestibule.

The present mRNA and protein results obtained from mice cochlea regarding *Cnrip1* corroborate the transcriptome data in which a decrease in the expression level in spiral ganglion (SGN) neurons from age P1 (neonates) to age P30 is observed (adults). This fact favors the hypothesis initially formulated that the duplication-driven overexpression of a gene, specifically in adulthood when this expression should not be decreased, could be the underlying cause of hearing loss in the DFNA58 family.

The International Mouse Phenotyping Consortium, whose goal is to generate knockout mice for all genes, recently updated the phenotypic data for the *Cnrip1* gene knockout mouse (https://www.mousephenotype.org/data/genes/MGI:1917505/# order, accessed on February 2021). They performed a hearing assessment in mice, using ABR (auditory brainstem response), at around 14 weeks of age and did not observe significant differences when compared to wild animals, as well as any other phenotypic change. This is in agreement with the study carried out by Fin et. al. [64], who also did not observe phenotypic changes (in terms of eyes, brain, spinal cord, muscles, notochord, fins, pigmentation, swim bladder, mandible, intestine, and in the shape and size of specimens) in *Cnrip1a* and *Cnrip1b* double knockout zebrafish, although hearing in fish has not been evaluated. Thus, it remains to be determined whether there would be hearing impairment and at what age in the case of the overexpression of the Cnrip1 gene in the cochlea. However, there are no publications about it so far.

## 5. Conclusions

Our data support *Cnrip1* as the most significant candidate to play a role in hearing. Even though *PPP3R1* represented the second-best candidate for DFNA58, given that it has only exon 1 duplicated, it is unlikely to participate in the phenotype.

In addition, the findings from both mice (*Cnrip1*), where the expression of these genes was more closely present in the spiral ganglion neurons, and from zebrafish (*Cnrip1a*), suggest that the gene has a relevant function due to the expression levels in cells important to the hearing physiology. However, this function in fish is exercised in the HCs and, in mammals, in the spiral ganglion neurons, and may or may not be distinct. Due to the difficulty in anatomically locating the statoacoustic ganglion of zebrafish for dissection and RNA extraction, it is not possible to say precisely that the *Cnrip1a* gene does not also play an important role in these cells; however, its importance in hearing seems to have remained conserved.

## Data Availability

All date are available in the figrues and Supplementary Material.

## Supplementary Materials

The following supporting information can be downloaded at: https://www.mdpi.com/article/10.3390/genes13122274/s1,; Supplementary Table S1: List of Zebrafish ages, tissues used, and the amount of each one shown in parentheses; Supplementary Table S2: List of primers used for real-time quantitative PCR analyses; Supplementary Table S3: List of primers used to perform the amplification of the cDNA that was used as a template for the transcription of the sense and antisense RNA probes in the in situ hybridization experiments; Supplementary Table S4: Cycle Threshold (Ct) values of the different genes among the different zebrafish tissues under analysis; Supplementary Table S5: Relative quantification of each gene in the different tissues analyzed in zebrafish; In the last column, genes differentially expressed between hair cells and their surrounding cells, among those studied by us, are highlighted studied in zebrafish; Supplementary Table S6: RPKM values of the Li et al. [30] study performed with mice; Supplementary Table S7 Gene expression of literature data regarding genes and tissues studied in zebrafish. Supplementary Figure S1: Expression analysis (RT-qPCR) of the reference/control genes in the adult zebrafish tissues [84,85,86,87,88,89,90,91,92,93,94,95,96].

## References

[1] Mulrow C.D., Aguilar C., Endicott J.E., Velez R., Tuley M.R., Charlip W.S., Hill J.A. (1990). Association Between Hearing Impairment and the Quality of Life of Elderly Individuals. J. Am. Geriatr. Soc..

[2] Cacciatore F., Napoli C., Abete P., Marciano E., Triassi M., Rengo F. (1999). Quality of life determinants and hearing function in an elderly population: Osservatorio Geriatrico Campano Study Group. Gerontology.

[3] World Health Organization Deafness and Hearing Loss. https://www.who.int/health-topics/hearing-loss#tab=tab_1.

[4] Petit C., Levilliers J., Hardelin J.P. (2001). Molecular genetics of hearing loss. Annu. Rev. Genet..

[5] Batissoco A.C., Pedroso-Campos V., Pardono E., Sampaio-Silva J., Sonoda C.Y., Vieira-Silva G.A., da Silva de Oliveira Longati E.U., Mariano D., Hoshino A.C.H., Tsuji R.K. (2022). Molecular and genetic characterization of a large Brazilian cohort presenting hearing loss. Hum. Genet..

[6] Shearer E.A., Hildebrand M.S., Smith R.J., Adam M.P., Everman D.B., Mirzaa G.M., Pagon R.A., Wallace S.E. (2017). Hereditary Hearing Loss and Deafness Overview. GeneReviews^®^.

[7] Van Camp G., Smith R.J.H. Hereditary Hearing Loss Homepage. https://hereditaryhearingloss.org.

[8] Lezirovitz K., Braga M.C., Thiele-Aguiar R.S., Auricchio M.T., Pearson P.L., Otto P.A., Mingroni-Netto R.C. (2009). A novel autosomal dominant deafness locus (DFNA58) maps to 2p12-p21. Clin. Genet..

[9] Nicolson T. (2005). The genetics of hearing and balance in zebrafish. Annu. Rev. Genet..

[10] Whitfield T.T. (2002). Zebrafish as a model for hearing and deafness. J. Neurobiol..

[11] He Y., Bao B., Li H. (2017). Using zebrafish as a model to study the role of epigenetics in hearing loss. Expert Opin. Drug Discov..

[12] Xia W., Hu J., Mam J., Huang J., Wang X., Jiang N., Zhang J., Ma Z., Ma D. (2019). Novel TRRAP mutation causes autosomal dominant non-syndromic hearing loss. Clin. Genet..

[13] Harris J.A., Cheng A.G., Cunningham L.L., MacDonald G., Raible D.W., Rubel E.W. (2003). Neomycin-induced hair cell death and rapid regeneration in the lateral line of zebrafish (Danio rerio). JARO -J. Assoc. Res. Otolaryngol..

[14] Ma E.Y., Rubel E.W., Raible D.W. (2008). Notch signaling regulates the extent of hair cell regeneration in the zebrafish lateral line. J. Neurosci..

[15] Shen X., Liu F., Wang Y., Wang H., Mam J., Xia W., Zhang J., Jiang N., Sun S., Wang X. (2015). Down-regulation of msrb3 and destruction of normal auditory system development through hair cell apoptosis in zebrafish. Int. J. Dev. Biol..

[16] Sielemann K., Hafner A., Pucker B. (2020). The reuse of public datasets in the life sciences: Potential risks and rewards. PeerJ.

[17] Curty R.G., Crowston K., Specht A., Grant B.W., Dalton E.D. (2017). Attitudes and norms affecting scientists’ data reuse. PLoS ONE.

[18] Wade T.D. (2014). Traits and types of health data repositories. Heal. Inf. Sci. Syst..

[19] Wang B., Li R., Lu Z., Huang Y. (2020). Does comorbidity increase the risk of patients with covid-19: Evidence from meta-analysis. Aging (Albany. NY).

[20] Barrett T., Wilhite S.E., Ledoux P., Evangelista C., Kim I.F., Tomashevsky M., Marshall K.A., Phillippy K.H., Sherman P.M., Holko M. (2013). NCBI GEO: Archive for functional genomics data sets - Update. Nucleic Acids Res..

[21] Kodama Y., Shumway M., Leinonen R. (2012). The sequence read archive: Explosive growth of sequencing data. Nucleic Acids Res..

[22] Rustici G., Kolesnikov N., Brandizi M., Burdett T., Dylag M., Emam I., Farne A., Hastings E., Ison J., Keays M. (2013). ArrayExpress update-trends in database growth and links to data analysis tools. Nucleic Acids Res..

[23] Erickson T., Nicolson T. (2015). Identification of sensory hair-cell transcripts by thiouracil-tagging in zebrafish. BMC Genom..

[24] McDermott B.M., Baucom J.M., Hudspeth A.J. (2007). Analysis and functional evaluation of the hair-cell transcriptome. Proc. Natl. Acad. Sci. USA.

[25] Barta C.L., Liu H., Chen L., Giffen K.P., Li Y., Kramer K.L., Beisel K.W., He D.Z. (2018). RNA-seq transcriptomic analysis of adult zebrafish inner ear hair cells. Sci. Data.

[26] Liu H., Pecka J.L., Zhang Q., Soukup G.A., Beisel K.W., He D.Z.Z. (2014). Characterization of transcriptomes of cochlear inner and outer hair cells. J. Neurosci..

[27] Elkon R., Milon B., Morrison L., Shah M., Vijayakumar S., Racherla M., Leitch C.C., Silipino L., Hadi S., Weiss-Gayet M. (2015). RFX transcription factors are essential for hearing in mice. Nat. Commun..

[28] Cai T., Jen H.I., Kang H., Klisch T.J., Zoghbi H.Y., Groves A.K. (2015). Characterization of the transcriptome of nascent hair cells and identification of direct targets of the atoh1 transcription factor. J. Neurosci..

[29] Burns J.C., Kelly M.C., Hoa M., Morell R.J., Kelley M.W. (2015). Single-cell RNA-Seq resolves cellular complexity in sensory organs from the neonatal inner ear. Nat. Commun..

[30] Li Y., Liu H., Giffen K.P., Chen L., Beisel K.W., He D.Z.Z. (2018). Transcriptomes of cochlear inner and outer hair cells from adult mice. Sci. Data.

[31] Li C., Li X., Bi Z., Sugino K., Wang G., Zhu T., Liu Z. (2020). Comprehensive transcriptome analysis of cochlear spiral ganglion neurons at multiple ages. Elife.

[32] Sun S., Babola T., Pregernig G., So K.S., Nguyen M., Su S.M., Palermo A.T., Bergles D.E., Burns J.C., Müller U. (2018). Hair Cell Mechanotransduction Regulates Spontaneous Activity and Spiral Ganglion Subtype Specification in the Auditory System. Cell.

[33] Lezirovitz K., Vieira-Silva G.A., Batissoco A.C., Levy D., Kitajima J.P., Trouillet A., Ouyang E., Zebarjadi N., Sampaio-Silva J., Pedroso-Campos V. (2020). A rare genomic duplication in 2p14 underlies autosomal dominant hearing loss DFNA58. Hum. Mol. Genet..

[34] Baumgart M., Priebe S., Groth M., Hartmann N., Menzel U., Pandolfini L., Koch P., Felder M., Ristow M., Englert C. (2016). Longitudinal RNA-seq analysis of vertebrate aging identifies mitochondrial complex i as a small-molecule-sensitive modifier of lifespan. Cell Syst..

[35] Oosterhof N., Holtman I.R., Kuil L.E., van der Linde H.C., Boddeke E.W., Eggen B.J., van Ham T.J. (2017). Identification of a conserved and acute neurodegeneration-specific microglial transcriptome in the zebrafish. Glia.

[36] Hertzano R., Elkon R., Kurima K., Morrisson A., Chan S.L., Sallin M., Biedlingmaier A., Darling D.S., Griffith A.J., Eisenman D.J. (2011). Cell type-specific transcriptome analysis reveals a major role for Zeb1 and miR-200b in mouse inner ear morphogenesis. PLoS Genet..

[37] Einarsson R., Haden M., Diciolli G., Lim A., Mah-Ginn K., Aguilar K., Yazejian L., Yazejian B. (2012). Patch clamp recordings in inner ear hair cells isolated from zebrafish. J. Vis. Exp..

[38] Matsuzaki S., Hosoya M., Okano H., Fujioka M., Ogawa K. (2018). Expression pattern of EYA4 in the common marmoset (Callithrix jacchus) cochlea. Neurosci Lett..

[39] Busch-Nentwich E., Söllner C., Roehl H., Nicolson T. (2004). The deafness gene dfna5 is crucial for ugdh expression and HA production in the developing ear in zebrafish. Development.

[40] Schwarzer S., Asokan N., Bludau O., Kuscha V., Kaslin J., Hans S. (2020). Neurogenesis in the inner ear: The zebrafish statoacoustic ganglion provides new neurons from a Neurod/Nestin-positive progenitor pool well into adulthood. Development.

[41] Thisse B., Heyer V., Lux A., Alunni V., Degrave A., Seiliez I., Kirchner J., Parkhill J.P., Thisse C. (2004). Spatial and temporal expression of the zebrafish genome by large-scale in situ hybridization screening. Methods Cell Biol..

[42] Giffen K.P., Liu H., Kramer K.L., He D.Z. (2019). Expression of Protein-Coding Gene Orthologs in Zebrafish and Mouse Inner Ear Non-sensory Supporting Cells. Front. Neurosci..

[43] Maskell L.J., Qamar K., Babakr A.A., Hawkins T.A., Heads R.J., Budhram-mahadeo V.S. (2017). Essential but partially redundant roles for POU4F1/Brn-3a and POU4F2/Brn-3b transcription factors in the developing heart. Nat. Publ. Gr..

[44] Yousaf R., Ahmed Z.M., Giese A.P., Morell R.J., Lagziel A., Dabdoub A., Wilcox E.R., Riazuddin S., Friedman T.B., Riazuddin S. (2018). Modifier variant of METTL13 suppresses human GAB1-associated profound deafness. J. Clin. Investig..

[45] Pfaffl M.W. (2001). A new mathematical model for relative quantification in real-time RT–PCR. Nucleic Acids Res..

[46] Rassier G.T., Silveira T.L.R., Remião M.H., Daneluz L.O., Martins A.W.S., Dellagostin E.N., Ortiz H.G., Domingues W.B., Komninou E.R., Kütter M.T. (2020). Evaluation of qPCR reference genes in GH-overexpressing transgenic zebrafish (Danio rerio). Sci. Rep..

[47] Xu H., Li C., Zeng Q., Agrawal I., Zhu X., Gong Z. (2016). Genome-wide identification of suitable zebrafish Danio rerio reference genes for normalization of gene expression data by RT-qPCR. J. Fish. Biol..

[48] Krohs C., Bordeynik-Cohen M., Messika-Gold N., Elkon R., Avraham K.B., Nothwang H.G. (2021). Expression pattern of cochlear microRNAs in the mammalian auditory hindbrain. Cell Tissue Res..

[49] Thisse C., Thisse B. (2008). High-resolution in situ hybridization to whole-mount zebrafish embryos. Nat. Protoc..

[50] Hu S.S., Arnold A., Hutchens J.M., Radicke J., Cravatt B.F., Wager-Miller J., Mackie K., Straiker A. (2010). Architecture of cannabinoid signaling in mouse retina. J. Comp. Neurol..

[51] Smith T.H., Blume L.C., Straiker A., Cox J.O., David B.G., McVoy J.R., Sayers K.W., Poklis J.L., Abdullah R.A., Egertová M. (2015). Cannabinoid receptor-interacting protein 1a modulates CB1 receptor signaling and regulation. Mol. Pharmacol..

[52] Deng M., Yang H., Xie X., Liang G., Gan L. (2014). Comparative expression analysis of POU4F1, POU4F2 and ISL1 in developing mouse cochleovestibular ganglion neurons. Gene Expr. Patterns.

[53] Maier E.C., Saxena A., Alsina B., Bronner M.E., Whitfield T.T. (2014). Sensational placodes: Neurogenesis in the otic and olfactory systems. Dev. Biol..

[54] Bang P.I., Yelick P.C., Malicki J.J., Sewell W.F. (2002). High-throughput behavioral screening method for detecting auditory response defects in zebrafish. J. Neurosci. Methods.

[55] Kindt K.S., Sheets L., Wingert R.A. (2018). Transmission Disrupted: Modeling Auditory Synaptopathy in Zebrafish. Front. Cell Dev. Biol..

[56] Ghysen A., Dambly-chaudie C. (2004). Development of the zebrafish lateral line. Curr. Opin. Neurobiol..

[57] Haehnel-Taguchi M., Fernandes A.M., Böhler M., Schmitt I., Tittel L., Driever W. (2018). Projections of the diencephalospinal dopaminergic system to peripheral sense organs in larval zebrafish (Danio rerio). Front. Neuroanat..

[58] Rabbitts T.H. (2008). Chromosomal translocations in cancer. Biochim. Biophys. Acta - Rev. Cancer.

[59] Guarnerio J., Bezzi M., Jeong J.C., Paffenholz S.V., Berry K., Naldini M.M., Lo-Coco F., Tay Y., Beck A.H., Pandolfi P.P. (2016). Oncogenic Role of Fusion-circRNAs Derived from Cancer-Associated Chromosomal Translocations. Cell.

[60] Barrett S.P., Wang P.L., Salzman J. (2015). Circular RNA biogenesis can proceed through an exon-containing lariat precursor. Elife.

[61] Jeck W.R., Sorrentino J.A., Wang K., Slevin M.K., Burd C.E., Liu J., Marzluff W.F., Sharpless N.E. (2013). Circular RNAs are abundant, conserved, and associated with ALU repeats. RNA.

[62] Liang D., Wilusz J.E. (2014). Short intronic repeat sequences facilitate circular RNA production. Genes Dev..

[63] Zhang X.O., Bin Wang H., Zhang Y., Lu X., Chen L.L., Yang L. (2014). Complementary sequence-mediated exon circularization. Cell.

[64] Fin L., Bergamin G., Steiner R.A., Hughes S.M. (2017). The Cannabinoid Receptor Interacting Proteins 1 of zebrafish are not required for morphological development, viability or fertility. Sci. Rep..

[65] Elphick M.R. (2012). The evolution and comparative neurobiology of endocannabinoid signalling. Philos. Trans. R. Soc. B Biol. Sci..

[66] Guggenhuber S., Romo-Parra H., Bindila L., Leschik J., Lomazzo E., Remmers F., Zimmermann T., Lerner R., Klugmann M., Pape H.C. (2016). Impaired 2-AG signaling in hippocampal glutamatergic neurons: Aggravation of anxiety-like behavior and unaltered seizure susceptibility. Int. J. Neuropsychopharmacol..

[67] Niehaus J.L., Liu Y., Wallis K.T., Egertová M., Bhartur S.G., Mukhopadhyay S., Shi S., He H., Selley D.E., Howlett A.C. (2007). CB1 cannabinoid receptor activity is modulated by the cannabinoid receptor interacting protein CRIP 1a. Mol. Pharmacol..

[68] Kreitzer A.C., Regehr W.G. (2001). Cerebellar depolarization-induced suppression of inhibition is mediated by endogenous cannabinoids. J. Neurosci..

[69] Wilson R.I., Kunos G., Nicoll R.A., Francisco S. (2001). Presynaptic Specificity of Endocannabinoid Signaling in the Hippocampus. Neuron.

[70] Sjöström P.J., Turrigiano G.G., Nelson S.B. (2003). Neocortical LTD via Coincident Activation of Presynaptic NMDA and Cannabinoid Receptors. Neuron.

[71] Galvan A., Frullanti E., Anderlini M., Manenti G., Noci S., Dugo M., Ambrogi F., De Cecco L., Spinelli R., Piazza R. (2013). Gene expression signature of non-involved lung tissue associated with survival in lung adenocarcinoma patients. Carcinogenesis.

[72] Jayaraman T., Marks A.R. (2000). Calcineurin Is Downstream of the Inositol 1, 4, 5-Trisphosphate Receptor in the Apoptotic and Cell Growth Pathways *. J. Biol. Chem..

[73] Orrenius S., Zhivotovsky B., Nicotera P. (2003). Regulation of cell death: The calcium-apoptosis link. Nat. Rev. Mol. Cell Biol..

[74] Minami S.B., Yamashita D., Schacht J., Miller J.M. (2004). Calcineurin activation contributes to noise-induced hearing loss. J. Neurosci. Res..

[75] Sano K., Takai Y., Yamanishi J., Nishizuka Y. (2013). A Role of Calcium-activated Phospholipid-dependent Protein Kinase in Human Platelet Activation. J. Biol. Chem..

[76] Tyers M., Rachubinski R.A., Stewart M.I., Varrichio A.M., Shorr R.G., Haslam R.J., Harley C.B. (1988). Molecular cloning and expression of the major protein kinase C substrate of platelets. Nature.

[77] Winstel R., Freund S., Krasel C., Hoppe E., Lohset M.J. (1996). Protein kinase cross-talk: Membrane targeting of the receptor kinase by protein kinase C. Proc. Natl. Acad. Sci. USA.

[78] García-Sáinz J.A., Vázquez-Prado J., del Carmen Medina L. (2000). Alpha 1-adrenoceptors: Function and phosphorylation. Eur J. Pharmacol..

[79] Cmarik J.L., Hegamyer G., Gerrard B., Dean M., Colburn N.H. (2000). cDNA cloning and mapping of mouse pleckstrin (Plek), a gene upregulated in transformation-resistant cells. Genomics.

[80] Kitajiri S., Fukumoto K., Hata M., Sasaki H., Katsuno T., Nakagawa T., Ito J., Tsukita S., Tsukita S. (2004). Radixin deficiency causes deafness associated with progressive degeneration of cochlear stereocilia. J. Cell Biol..

[81] Hilgert N., Smith R.J.H., Van Camp G. (2009). Function and expression pattern of nonsyndromic deafness genes. Curr. Mol. Med..

[82] Salazar-Silva R., Dantas V.L.G., Alves L.U., Batissoco A.C., Oiticica J., Lawrence E.A., Kawafi A., Yang Y., Nicastro F.S., Novaes B.C. (2021). NCOA3 identified as a new candidate to explain autosomal dominant progressive hearing loss. Hum. Mol. Genet..

[83] Oltrabella F., Melgoza A., Nguyen B., Guo S. (2017). Role of the endocannabinoid system in vertebrates: Emphasis on the zebrafish model. Dev. Growth Differ..

[84] BGEE Database. https://bgee.org/.

[85] Blasiole B., Canfield V.A., Vollrath M.A., Huss D., Mohideen M.-A.P., Dickman J.D., Cheng K., Fekete D.M., Levenson R. (2006). Separate Na,K-ATPase genes are required for otolith formation and semicircular canal development in zebrafish. Dev. Biol..

[86] Breuer M., Guglielmi L., Zielonka M., Hemberger V., Kölker S., Okun J.G., Hoffmann G.F., Carl M., Sauer S.W., Opladen T. (2019). QDPR homologues in Danio rerio regulate melanin synthesis, early gliogenesis, and glutamine homeostasis. PLoS ONE.

[87] Cheng W., Guo L., Zhang Z., Soo H.M., Wen C., Wu W., Peng J. (2006). HNF factors form a network to regulate liver-enriched genes in zebrafish. Dev. Biol..

[88] Gesemann M., Lesslauer A., Maurer C.M., Schönthaler H.B., Neuhauss S.C.F. (2010). Phylogenetic analysis of the vertebrate Excitatory/Neutral Amino Acid Transporter (SLC1/EAAT) family reveals lineage specific subfamilies. BMC Evol. Biol..

[89] Hammond D.R., Udvadia A.J. (2010). Cabin1 expression suggests roles in neuronal development. Dev. Dyn..

[90] Le T.H., Duy P.Q., An M., Talbot J., Iyer C.C., Wolman M., Beattie C.E. (2017). HuD and the Survival Motor Neuron Protein Interact in Motoneurons and Are Essential for Motoneuron Development, Function, and mRNA Regulation. J. Neurosci..

[91] Hosoya M., Fujioka M., Ogawa K., Okano H. (2016). Distinct Expression Patterns Of Causative Genes Responsible For Hereditary Progressive Hearing Loss In Non-Human Primate Cochlea. Sci. Rep..

[92] Lu F.-I., Thisse C., Thisse B. (2011). Identification and mechanism of regulation of the zebrafish dorsal determinant. Proc. Natl. Acad. Sci. USA.

[93] Rico E.P., de Oliveira D.L., Rosemberg D.B., Mussulini B.H., Bonan C.D., Dias R.D., Wofchuk S., Souza D.O., Bogo M.R. (2010). Expression and functional analysis of Na+-dependent glutamate transporters from zebrafish brain. Brain Res. Bull..

[94] Sato T., Hamaoka T., Aizawa H., Hosoya T., Okamoto H. (2007). Genetic Single-Cell Mosaic Analysis Implicates ephrinB2 Reverse Signaling in Projections from the Posterior Tectum to the Hindbrain in Zebrafish. J. Neurosci..

[95] Schönberger J., Wang L., Shin J.T., Kim S.D., Depreux F.F.S., Zhu H., Zon L., Pizard A., Kim J.B., A MacRae C. (2005). Mutation in the transcriptional coactivator EYA4 causes dilated cardiomyopathy and sensorineural hearing loss. Nat. Genet..

[96] Wang L., Sewell W.F., Kim S.D., Shin J.T., MacRae C.A., Zon L.I., Seidman J.G., Seidman C.E. (2008). Eya4 regulation of Na+/K+-ATPase is required for sensory system development in zebrafish. Development.

