# Supplementary material for: New Insights into the Identity of the DFNA58 Gene
Source: Genes (Basel). 2022 Dec 2;13(12):2274. doi: 10.3390/genes13122274 (PMC9777997; doi:10.3390/genes13122274)
Supplement: Supplementary file 1 [file genes-13-02274-s001.zip › genes-2008562-supplementary.pdf]

Supplementary Table S1: List of Zebrafish ages, tissues used, and the amount of each one shown in parentheses.

Samples for RNA extraction		
**Technique**	**Specimen age**	**Tissue and quantity used**
in situ Hybridization	5 dpf	pool of 80 larvae
	1 year	whole organism (1)
RT-qPCR	4 months	IE (2)
	2-3 years	IE (1)
		pool of Utricule (7) + Lagena (5)
		Heart (2)
		Swimming bladder (2)

Supplementary Table S2: List of primers used for real-time quantitative PCR analyses.

Gene	Sequence	Size	Reason of selection	Reference
Ppp3r1a	F: GCTGTTCATTTCCGAGGGG<br>R: TGCGAGCACATTTCCAGGG	84bp	candidate to DFNA58	Lezirovitz et al. 2020
Ppp3r1b	F: GTCAGATTCTGCACAAGGCG<br>R: TGTGAGCACATCTCCAAGGG	84bp	candidate to DFNA58	Lezirovitz et al. 2020
Plek	F: AGGTGCGATAGTCAAACAGGG<br>R: GCCCAGAGGATCATCTTCACC	142bp	candidate to DFNA58	Lezirovitz et al. 2020
Cnrip1b	F: CAGCCTGTTCAGGTCAGCATACA<br>R: ATGCTGTTGAAGCTGTTCCCC	109bp	candidate to DFNA58	Lezirovitz et al. 2020
Cnrip1a	F: CAACTACGACACTGAGGGCG<br>R: AGACGGTTTCGAACATCCCG	101bp	candidate to DFNA58	Lezirovitz et al. 2020
Elavl4	F: CAGAAGGAGCTGGAACAACTCT<br>R: GCGAGCCACCTGTGGGG	94bp	Important expression in the IE/ SAG neuronal marker	Schwarzer et al., 2020; Li et al., 2020
Gab1	F: TCGACTTGGACCCTGGGAAATCAA<br>R: TGCTGATCCACCAGCACATAGTCT	106bp	Important expression in the inner ear- IE marker / Associated with DFNB26	Yousaf et al., 2018
Mettl13	F: AGACATGCAGGCTGTCCAATCAGA<br>R: AAGCACTGAGCCCTCTTCCAATCA	146bp	Important expression in the IE/ Associated with DFNM1	Yousaf et al., 2018
Gsdmea	F: ACTCCGTTTCTCCATCTTGGTC<br>R: AGCTTCGTCACTGCTCAGG	103bp	Important expression in the spiral ganglion/ Associated with DFNA5	Hosoya *et al., 2016*
Gsdmeb	F: TCTACAGTCAAACAACCACGAGA<br>R: AGGCACAACACTGGGATCAG	141bp	Important expression in the spiral ganglion/ Associated with DFNA5	Hosoya *et al., 2016*
Eya4	F: CCCACTGCCCCAAGTATGAA<br>R: GGACTGAATCCACTGCTGCT	131bp	Important expression in the spiral ganglion/ Associated with DFNA10	Matsuzaki et al., 2018
Pou4f1	F: GGACTCGTCAACAACGGACT<br>R: CACCCCGAGTTTGATCCTCC	108bp	Important expression in the spiral ganglion/ Sensory neuron marker in zebrafish	Lie t al., 2020
Slc1a3a	F: TGTCGTATCGGGAGGTGAAG<br>R: ACTGTCCAGAGCTGCCATTC	122bp	Important expression in the inner ear of Zebrafish and low in the surrounding tissues. Mice that are null for this gene have vestibular phenotype of late-onset circling behavior at 7 months of age.	Schraven et al., 2012;; Thisse et al., 2004
Rpl13	F: AGCTGCGCTATTCCAAGAAGA<br>R: ACTGCGATCTTGCTCTCAACG	79bp	Endogenous Gene/Housekeeping/Reference	Rassier et al., 2020
Eef1a1	F: CTGGAGGCCAGCTCAAACAT<br>R: ATCAAGAAGAGTAGTACCGCTAGCATTAC	176bp	Endogenous Gene/Housekeeping/Reference	Rassier et al., 2020
Rps18	F: GGGAAATACAGCCAGGTCCTT<br>R: CACGGTGAGCCCTGATCTTC	88bp	Endogenous Gene/Housekeeping/Reference	Rassier et al., 2020
Actb2	F: CGTGCTGTCTTCCCATCCA<br>R: TCACCAACGTAGCTGTCTTTCTG	86bp	Endogenous Gene/Housekeeping/Reference	Rassier et al., 2020
Sep15	F: CCTGTGAGCTTCTGGGTCAG<br>R: TCAAGGATGGCTCCTGGGTA	115bp	Endogenous Gene/Housekeeping/Reference	Xu et al., 2016
Metap1	F: CCATGGCCCGGGTACAGATA<br>R: CTCCGACATCCCCAAAGGATGA	123bp	Endogenous Gene/Housekeeping/Reference	Xu et al., 2016
Gapdh	F: AATTCTGGGATACACGGAGCACCA<br>R: TCAGGTCACATACACGGTTGCTGT	167bp	Endogenous Gene/Housekeeping/Reference	Yousaf et al., 2018; Rassier et al., 2020

Supplementary Table S3: Cycle Threshold (Ct) values of the different genes among the different zebrafish tissues under analysis.

	**Mean of C_t ± DP values among the different tissues**					
Gene	**LG+UT**	**BRN**	**HRT**	**SWB**	**IE**	**SD**
Ppp3r1a	**28.19±0.81**	**21.76±0.00**	**26.70±0.10**	**17.84±0.32**	**19.84±0.04**	
Ppp3r1b	**28.60±0.04**	**21.65±0.05**	**23.83±0.09**	**21.85±0.14**	**25.29±0.07**	
Cnrip1b	**33.44±0.68**	**26.38±0.06**	**34.02±0.24**	**28.18±0.32**	**33.97±0.30**	
Cnrip1a	**28.87±027**	**23.05±0.12**	**28.18±0.10**	**28.09±0.24**	**28.15±0.31**	
Plek	**29.95±0.25**	**26.83±0.19**	**26.53±0.12**	**27.34±0.08**	**26.42±0.10**	
Slc1a3a	**25.21±0.11**	**24.51±0.02**	**25.44±0.05**	**23.66±0.06**	**24.59±0.09**	
Eya4	**29.94±0.53**	**32.78±0.12**	**30.83±0.46**	**32.10±0.40**	**29.42±0.34**	
Elavl4	**27.88±0.12**	**21.45±0.16**	**29.30±0.18**	**28.37±0.17**	**26.35±0.12**	
GsmdeB/dfna5b	**30.28±0.16**	**28.92±0.37**	**28.32±0.32**	**26.19±0.19**	**27.03±0.06**	
GsmdeA/dfna5a	**30.42±0.12**	**28.27±0.02**	**27.29±0.13**	**26.61±0.24**	**27.20±0.03**	
Pou4f1	**24.72±0.26**	**24.70±0.19**	**undet.**	**26.33±0.11**	**26.97±0.15**	
Gab1	**30.13±0.31**	**27.14±0.05**	**26.28±0.03**	**23.05±0.12**	**27.31±0.15**	
Mettl13	**29.21±0.16**	**27.78±0.34**	**29.04±0.5**	**22.95±0.08**	**29.38±0.16**	
Gapdh	**26.42±0.30**	**21.87±0.07**	**14.97±0.12**	**16.09±0.24**	**18.58±0.04**	4.50
Actb2	**21.08±0.30**	**17.28±0.04**	**17.49±0.12**	**15.67±0.22**	**17.17±0.03**	1.94
Rpl13	**22.84±0.09**	**19.82±0.30**	**18.65±0.15**	**17.84±0.32**	**19.84±0.04**	1.83

LG+UT=Lagena+Utricle. BRN=Brain. HRT=Heart. SWB=swim bladder. IE=inner ear

SD=standard deviation

Supplementary Table S4: List of primers used to perform the amplification of the cDNA that was used as a template for the transcription of the sense and antisense RNA probes in the in situ hybridization experiments, and the size in bp of the expected PCR fragments, as well as where each primer anneals. The bold lowercase letters in the forward and reverse primers correspond to the T7 promoter sequence. All primers anneal in the untranslated regions (UTR), 5' and 3'.

**Genes**	**Probes**	**Primer name**	**Sequence (5'-3')**	**Anneal to**	**Fragment Size**
Cnrip1a	anti-sense	CNRIP1a-ZF-antisense-F	AAACAGCTTCCTGGGAGCATC	Exon 1	679 bp
		CNRIP1a-ZF-antisense-R	taattacgactcactatagggagATACCCTGACGGATCGCTTG	Exon 3	
	sense (control)	CNRIP1a-ZF-sense-F	taattacgactcactatagggagAAACAGCTTCCTGGGAGCATC	Exon 1	679 bp
		CNRIP1a-ZF-sense-R	ATACCCTGACGGATCGCTTG	Exon 3	

Supplementary Table S5: Relative quantification of each gene in the different tissues analyzed in zebrafish. In the last column. genes differentially expressed between hair cells and their surrounding cells. among those studied by us. are highlighted studied in zebrafish.

	**Zebrafish adult (11-13 months)**		**Zebrafish adult (3 months)**	**Zebrafish adult (~36 months)**	**Zebrafish adult (11-13 months)**
**Ref**	**Barta et al.. 2018**		**Oosterhof et al.. 2017**	**Baumgart et al.. 2016**	**Barta et al.. 2018**
**Gene**	**Cell/tissue**				**Fold difference (Log2)**
	**HC**	**ncSC**	**Microglial**	**Liver**	**CC/ncSC**
Plek	2.65±2.96	1.4±0.43	96.19±19.13	0.94±0.67	------
Ppp3r1a	6.99±0.99	3.75±0.51	8.93±4.60	2.19±0.26	------
Ppp3r1b	10.63±0.77	12.80±0.14	20.96±1.06	6.85±0.44	------
Cnrip1a	**52.01±1.17**	**0.92±0.27**	**0.32±0.32**	**0.07±0.04**	**5.83**
Cnrip1b	0	0	0.00	0.02±0.03	------
Slc1a3a	**62.12±2.58**	**5.17±0.5**	40.56±3.32	32.43±3.02	**3.59**
Eya4	**140.07±3.29**	**2.38±0.27**	0.10±0.17	0.33±0.11	**5.88**
Elavl4	4.00±0.18	2.29±0.6	1.14±0.17	0.02±0.01	------
Gsmdea	0	0.2±0.03	2.55±1.01	4.79±1.47	0.20
Gsmdeb	5.5±0.2	4.85±0.56	0.24±0.26	0.00	------
Pou4f1	**35.65±2.45**	**0.11±0.02**	0.09±0.1	0	**8.30**
Gab1	6.98±0.13	8.94±1.24	8.57±3.03	2.87±0.61	------
Mettl13	0.37±0.07	0.98±0.03	1.38±0.89	0.42±0.1	**1.39**

Supplementary Table S6: RPKM values of the LI et al. (2018) study performed with mice.

Mouse (P28-P35)						
**Ref**	**Li et al.. 2018**					
Gene	**IHC**	**OHC**	**DEG (IHC/OHC)**	**Deiters**	**Pilar**	**DEG (Deiters/Pilar)**
Plek	0.00	0.15±0.06	---	1.32±0.14	1.97±0.13	---
Ppp3r1	17.34±1.55	9.95±0.87	0.80	29.84±2.05	14.63±1.25	1.03
Cnrip1	0.11±0.06	0.23±0.04	-1.06	0.85±0.19	0.61±0.13	0.48
Slc1a3	**131.02±1.47**	**7.67±0.25**	**2.02**	**5.53±0.34**	**213.19±1.22**	**-5.27**
Eya4	7.24±0.13	12.32±0.82	-0.77	5.63±0.44	9.25±0.42	-0.72
Elavl4	0.61±0.07	0.8±0.09	-0.39	1.88±0.1	0.82±0.18	1.20
Gsmde/Dfna5	0.62±0.1	0.27±0.05	1.20	0.65±0.09	0.95±0.15	-0.55
Pou4f1	0.00	0.00	---	0.02±0.03	0.15±0.05	---
Gab1	12.36±0.5	9.18±0.65	0.43	41.04±1.15	28.14±0.90	0.54
Mettl13	0.79±0.02	1.78±0.21	-1.17	2.36±0.28	2.15±0.20	0.13

Supplemntary Table S7: Gene expression of literature data regarding genes and tissues studied in zebrafish.

Gene expression								
**Gene**	**Age studied**	**Organ/Tissue**						**Ref.**
			**Brain**	**IE**	**Eye**	**Heart**	**Swim bladder**	
Cnrip1a ENSDARG00000037337	10 hpf	whole larval body						Fin et al., 2017
	?	**Heart**				X		BGEE database
	?	**Swim bladder**					X	BGEE database
	?	**Inner Ear**			X			BGEE database
	?	**Brain**	X					BGEE database
	24-60 hpf	**Telencephalon**	X					Fin et al., 2017
	24-60 hpf	**anterior and postoptic commissure. pineal gland**	X					Fin et al., 2017
	24-60 hpf	**hindbrain**	X					Fin et al., 2017
	24-60 hpf	**spinal cord**						Fin et al., 2017
	24-60 hpf	**retinal ganglion cells. other ganglia of the head**	X					Fin et al., 2017
	?	**Inner Ear**		X				BGEE database
	24-60 hpf	**cnrip1a regions of postmitotic neurons**						Fin et al., 2017
	24-60 hpf	**olfactory receptors**						Fin et al., 2017
Cnrip1b *ENSDARG00000037337*	10 hpf	whole larval body						Fin et al., 2017
	?	**Heart**				X		BGEE database
	?	**Swim bladder**					X	BGEE database
	?	**Eye**		X				BGEE database
	?	**Brain**	X					BGEE database
		**diffuse spinal cord**	X		X	Fin et al., 2017		[51]
	24-48 hpf	**somites and pectoral fins**	X		X			Fin et al., 2017
Ppp3r1a ENSDARG00000092659	?	**Heart**				X		BGEE database
	?	**Swim bladder**					X	BGEE database
	?	**Eye**		X				BGEE database
	?	**Brain**	X					BGEE database
	72-96 hpf	**Epiphase and olfactory bulb**	X					Hammond & Udvadia, 2010
Ppp3r1b *ENSDARG00000069360*	?	**Heart**				X		BGEE database
	?	**Swim bladder**					X	BGEE database
	?	**Eye**		X				BGEE database
	72-96 hpf	**Brain**	X					BGEE database
		**Liver**				Hammond & Udvadia, 2010		[78]

		**optic tectum**	**X**			Hammond & Udvadia, 2010		[78]
	**72-96 hpf**	**olfactory bulb**	**X**					Hammond & Udvadia, 2010
Plek *ENSDARG00000104325*	?	**Heart**				**X**		BGEE database
	?	**Swim bladder**					**X**	BGEE database
	?	**Eye**		**X**				BGEE database
	?	**Brain**	**X**					BGEE database

Gene expression

**Gene**	**Age studied**	**Organ/Tissue**						**Ref.**
			**Cérebro**	**IE**	**Eye**	**Heart**	**Swim bladder**	
Elavl4 ENSDARG00000045639 Marcador pan-neuronal	30-42 hpf	**Heart**				**X**		BGEE database
	?	**Swim bladder**					**X**	BGEE database
	?	**Eye**		**X**				BGEE database
	all	**statoacoustic ganglion**	**X**	**X**				Schwarzer et al., 2020
	24-60 hpf	**Brain**	**X**					Hao et al., 2017
Gsdmeb (Dfna5) ENSDARG00000040485	22-24 hpf	**Neural crest. olfactory placode**	**X**					Busch-Nentwich et al., 2003
	?	**Eye**		**X**				BGEE database
	?	**Brain**	**X**					BGEE database
	?	**Swim bladder**					**X**	BGEE database
	30-42 hpf	**Heart**				**X**		Thisse & Thisse, 2004
	48-60 hpf	**Otic vesicle and semicircular canal**		**X**				Blasiole et al., 2006
	48-90 hpf	**Semicircular canal**		**X**				Blasiole et al., 2006
Gsdmea (Dfna5) ENSDARG00000086762	?	**Swim bladder**					**X**	BGEE database
	?	**Brain**	**X**					BGEE database
	?	**Heart**				**X**	**X**	BGEE database
	5-6 dpf	**Neuromasts**						Jian et al., 2014
Eya4 ENSDARG00000012397	Adult > 90 dpf	**Heart**				**X**		Schonberger et al.. 2005
	24-96 hpf / Adult > 60 dpf	**Anterior and posterior macula**		**X**				Wang et al.. 2008
	72-96 hpf / Adult > 60 dpf	**Lateral crista**		**X**				Wang et al.. 2008
	24-96	**Neuromast**						Schonberger et al.. 2005
	14-19 hpf	**Otic placode**						Wang et al.. 2008
	48-60 hpf / Adult > 60 dpf	**Otic vesicle**		**X**				Wang et al.. 2008
	24-42 hpf / Adult > 60 dpf	**SAG**		**SAG**				Wang et al.. 2008

	?	**Swim bladder**					X	BGEE database
Gab1 *ENSDARG00000037018*	?	**Swim bladder**					X	BGEE database
	?	**Heart**				X	X	BGEE database
	12-48 hpf	**Brain and otic vesicle**	X	X				Yousaf et al., 2018
Mettl13	?	**Swim bladder**					X	BGEE database
	12-48 hpf	**Brain and otic vesicle**	X	X				Yousaf et al., 2018
Slc1a3a	72-96	**Brain**	X					Breuer et al., 2019
	72-96	**Glial cell**	SNC					Gesemann et al., 2010
	5-6 dpf	**Lateral line**						Thisse & Thisse, 2004
	5-6 dpf	**Glial cells**	SNC					Gesemann et al., 2010
	19-60	**Otic vesicle**		X				Thisse & Thisse, 2004
	19-60	**Spinal cord**	SNC					Thisse & Thisse, 2004
	Adult > 90 dpf	**Cerebellum**	X					Rico et al., 2010
	Adult > 90 dpf	**Liver**						Cheng et al., 2006
	Adult > 90 dpf	**Brain**	X					Rico et al., 2010
Pou4f1	16 hpf - Adult > 90 dpf	**Brain**	X					Sato et al., 2007
	32-72	**Eye**		X				Sato et al., 2007
	52hpf-77hpf	**Otic Vesicle**		X				Sato et al., 2007
	24–72	**Heart**				X		Maskell et al., 2017

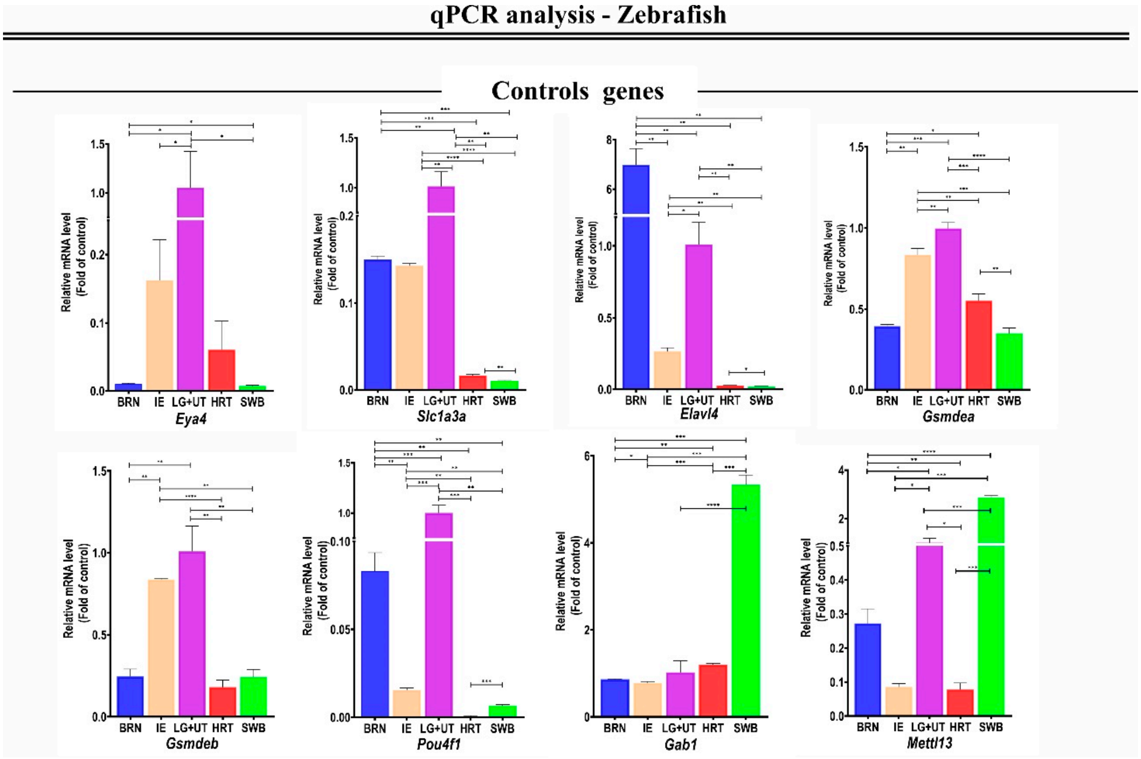

Supplementary Figure S1: Expression analysis (RT-qPCR) of the reference/control genes in the adult zebrafish tissues.

ADDITIONAL REFERENCES

BGEE database - https://bgee.org/

Blasiole B, Canfield VA, Vollrath MA, Huss D, Mohideen MA, Dickman JD, Cheng KC, Fekete DM, Levenson R. Separate Na,K-ATPase genes are required for otolith formation and semicircular canal development in zebrafish. Dev Biol. 2006 Jun 1;294(1):148-60. doi: 10.1016/j.ydbio.2006.02.034.

Breuer, M.; Guglielmi, L.; Zielonka, M.; Hemberger, V.; Kölker, S.; Okun, J.G.; Hoffmann, G.F.; Carl. M.; Sauer, S.W.; Opladen, T. QDPR homologues in Danio rerio regulate melanin synthesis, early gliogenesis, and glutamine homeostasis. PLoS One., 2019, 14, e0215162. doi: 10.1371/journal.pone.0215162. eCollection 2019.

Cheng, W.; Guo, L.; Zhang, Z.; Soo, H.M.; Wen, C.; Wu, W.; Peng, J. HNF factors form a network to regulate liver-enriched genes in zebrafish. Dev Biol., 2006, 294, 482-96. doi: 10.1016/j.ydbio.2006.03.018.

Gesemann, M.; Lesslauer, A.; Maurer, C.M.; Schönthaler, H.B.; Neuhauss, S.C. Phylogenetic analysis of the vertebrate excitatory/neutral amino acid transporter (SLC1/EAAT) family reveals lineage specific subfamilies. BMC Evol Biol., 2010, 10, 117. doi: 10.1186/1471-2148-10-117.

Hammond, D.R.; Udvadia, A.J. Cabin1 expression suggests roles in neuronal development. Dev Dyn., 2010, 239, 2443-51, doi: 10.1002/dvdy.22367.

Hao le T, Duy PQ, An M, Talbot J, Iyer CC, Wolman M, Beattie CE. HuD and the Survival Motor Neuron Protein Interact in Motoneurons and Are Essential for Motoneuron Development, Function, and mRNA Regulation. J Neurosci., 2017, 37, 11559-11571. doi: 10.1523/JNEUROSCI.1528-17.2017.

Hosoya M, Fujioka M, Ogawa K, Okano H. Distinct Expression Patterns Of Causative Genes Responsible For Hereditary Progressive Hearing Loss In Non-Human Primate Cochlea. Sci Rep. 2016 Feb 26;6:22250. doi: 10.1038/srep22250.

Lu FI, Thisse C, Thisse B. Identification and mechanism of regulation of the zebrafish dorsal determinant. Proc Natl Acad Sci U S A. 2011 Sep 20;108(38):15876-80. doi: 10.1073/pnas.1106801108.

Rico, E.P.; de Oliveira, D.L.; Rosemberg, D.B.; Mussulini, B.H.; Bonan, C.D.; Dias, R.D.; Wofchuk, S.; Souza, D.O.; Bogo, M.R. Expression and functional analysis of Na(+)-dependent glutamate transporters from zebrafish brain. Brain Res Bull., 2010, 81, 517-23. doi: 10.1016/j.brainresbull.2009.11.011.

Sato, T.; Hamaoka, T.; Aizawa, H.; Hosoya, T.; Okamoto, H. Genetic single-cell mosaic analysis implicates ephrinB2 reverse signaling in projections from the posterior tectum to the hindbrain in zebrafish. J Neurosci., 2007, 27, 5271-9. doi: 10.1523/JNEUROSCI.0883-07.2007.

Schönberger, J.; Wang, L.; Shin, J.T.; Kim, S.D.; Depreux, F.F.; Zhu, H.; Zon, L.; Pizard, A.; Kim, J.B.; Macrae, C.A.; Mungall, A.J.; Seidman, J.G.; Seidman, C.E. Mutation in the transcriptional coactivator EYA4 causes dilated cardiomyopathy and sensorineural hearing loss. Nat Genet., 2005, 37, 418-22. doi: 10.1038/ng1527.

Wang, L.; Sewell, W.F.; Kim, S.D.; Shin, J.T.; MacRae, C.A.; Zon, L.I.; Seidman, J.G.; Seidman, C.E. Eya4 regulation of Na+/K+-ATPase is required for sensory system development in zebrafish. Development., 2008, 135, 3425-34. doi: 10.1242/dev.012237.
